# Coplanar Dimeric Acceptors with Bathochromic Absorption and Torsion‐Free Backbones through Precise Fluorination Enabling Efficient Organic Photovoltaics with 18.63% Efficiency

**DOI:** 10.1002/advs.202410826

**Published:** 2025-01-20

**Authors:** Wei Liu, Weiwei Wu, Aleksandr A. Sergeev, Jia Yao, Yuang Fu, Chung Hang Kwok, Ho Ming Ng, Chunliang Li, Xiaojun Li, Sai Ho Pun, Huawei Hu, Xinhui Lu, Kam Sing Wong, Yongfang Li, He Yan, Han Yu

**Affiliations:** ^1^ Guangdong‐Hong Kong Joint Laboratory for Carbon Neutrality Jiangmen Laboratory of Carbon Science and Technology Jiangmen Guangdong 529199 P. R. China; ^2^ Department of Chemistry Hong Kong Branch of Chinese National Engineering Research Center for Tissue Restoration and Reconstruction Hong Kong University of Science and Technology Clear Water Bay Kowloon Hong Kong 999077 P. R. China; ^3^ Department of Physics William Mong Institute of Nano Science and Technology The Hong Kong University of Science and Technology Clear Water Bay Kowloon Hong Kong 999077 P. R. China; ^4^ Department of Physics Chinese University of Hong Kong Hong Kong New Territories 999077 P. R. China; ^5^ Beijing National Laboratory for Molecular Sciences CAS Key Laboratory of Organic Solids Institute of Chemistry Chinese Academy of Sciences Beijing 100190 P. R. China; ^6^ State Key Laboratory for Modification of Chemical Fibers and Polymer Materials College of Materials Science and Engineering Donghua University Shanghai 201620 P. R. China; ^7^ Hong Kong University of Science and Technology‐Shenzhen Research Institute No. 9, Yuexing 1st RD, Hi‐tech Park, Nanshan Shenzhen 518057 P. R. China; ^8^ Hong Kong University of Science and Technology Fok Ying Tung Research Institute S&T Building, Nansha IT Park Guangzhou City 511458 P. R. China; ^9^ Department of Applied Biology and Chemical Technology The Hong Kong Polytechnic University Hung Hom Kowloon Hong Kong 999077 P. R. China

**Keywords:** coplanarity, fluorination, giant dimeric acceptors, intramolecular charge transfer, organic solar cells

## Abstract

Giant dimeric acceptors (GDAs), a sub‐type of acceptor materials for organic solar cells (OSCs), have garnered much attention due to the synergistic advantages of their monomeric and polymeric acceptors, forming a well‐defined molecular structure with a giant molecular weight for high efficiency and stability. In this study, for the first time, two new GDAs, DYF‐V and DY2F‐V are designed and synthesized for OSC operation, by connecting one vinylene linker with the mono‐/di‐fluorinated end group on two Y‐series monomers, respectively. After fluorination, both DYF‐V and DY2F‐V exhibit bathochromic absorption and denser packing modes due to the stronger intramolecular charge transfer effect and torsion‐free backbones. Through precise fluorination, the DYF‐V‐based devices exhibit the highest performance of 18.63% among the GDA‐based OSCs, outperforming its non‐fluorinated counterpart, DY‐V‐based ones (16.53%). Theoretical and morphological results demonstrate that proper fluorination in DYF‐V‐based devices strengthens intra/intermolecular interactions for enhanced crystallinity, superior phase segregation, and less energy disorder, which is beneficial for fast exciton dissociation, rapid carrier transport, and suppressed charge recombination. The work demonstrates that proper fluorination on GDAs with rigid coplanar backbones is effective for broader photon harvesting, stronger packing, and robust stability in GDA‐based OSCs.

## Graphical abstract

A synergistic strategy of fluorinated end groups and vinylene linkers is employed for the strengthened intra/intermolecular interactions and bathochromic absorption of giant dimeric acceptors (GDAs). Compared to DY‐V (PCE = 16.53%), DYF‐V delivers a vastly improved performance with a PCE of 18.63% and a fill factor of 78.56%, both of which are the highest values among the GDA‐based organic solar cells.

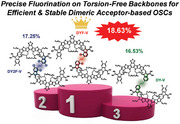

## 1 Introduction

Organic solar cells (OSCs)^[^ ^1^ ^]^ are considered one of the most promising solar technologies^[^ ^2^ ^]^ due to their portability, device transparency, low cost, and facile roll‐to‐roll manufacture.^[^ ^3^ ^]^ Recently, the advent of Y‐series small‐molecule acceptors (SMAs), which possess remarkable light absorption and charge transport properties, has resulted in a significant increase in the power conversion efficiencies (PCEs) of over 20% for OSCs.^[^ ^4^ ^]^ However, most SMA‐based OSCs suffer from poor operational stability due to the degradation of bulky heterojunction active layers^[^ ^5^ ^]^ under external stress, including thermal/mechanical stress and light illumination,^[^ ^6^ ^]^ which are seriously exacerbated by the rapid diffusion of SMAs in devices.^[^ ^7^ ^]^ To this end, polymer acceptors (PAs) in all‐polymer solar cells (all‐PSCs) are developed by scientists, exhibiting greater miscibility with donor polymers with much lower diffusion, which imparts superior stability to the blend. However, optimizing crystallinity in all‐PSCs still maintains a challenge owing to the amorphism of PAs,^[^ ^8^ ^]^ which limits their progress.

To balance this trade‐off between the crystallinity of SMAs and the stability of PAs, giant dimeric acceptors (GDAs)^[^ ^9^ ^]^ are being developed as promising alternatives (Figure S1 and Table S1, Supporting Information). GDAs possess larger molecular masses than SMAs,^[^ ^10^ ^]^ which reduces molecular diffusion rates and enhances morphology stability. Meanwhile, the GDAs^[^ ^11^ ^]^ exhibit superior crystallinity but precise molecular weight compared to PAs,^[^ ^1^, ^12^ ^]^ resulting in higher electron mobility and greater ease of morphology control. Recent studies have shown that GDAs could simultaneously enhance the efficiency and stability of OSCs.^[^ ^13^ ^]^ Since GDA is a new sub‐type of acceptors, the performance‐structure relationship is still not clear enough. Therefore, a comprehensive understanding of design strategies for the monomer backbone, end‐group, and linker unit is necessary. One effective strategy is the halogenation onto the end groups of monomers, which strengthens the intramolecular charge transfer (ICT) effect for extended photon response and enhanced crystallinity for faster carrier transport.^[^ ^14^ ^]^ Another effective approach is to build up rigid coplanar backbones using a torsion‐free linker unit, e.g., vinylene, which could stabilize polymer conformation for less energy disorder, and facilitate better intramolecular conjugation and intermolecular packing.^[^ ^15^ ^]^ Therefore, it is expected that the synergistic strategy above can be utilized to construct GDAs with broader photon harvesting and denser interchain packing for photovoltaic devices with higher efficiency and stability.

In this paper, we, for the first time, synthesized two new GDAs, named DYF‐V and DY2F‐V, for systematic study in OSCs. The two novel GDAs were synthesized by connecting the mono‐/di‐fluorinated end group on two Y‐series monomers with one vinylene linker, respectively, for a stronger ICT effect and torsion‐free backbones. After fluorination, both DYF‐V and DY2F‐V exhibited bathochromic absorption and tighter packing modes in comparison with their non‐fluorinated counterpart, DY‐V. Through tailoring the fluorination degree, the PM6:DYF‐V‐based devices exhibit the highest PCE of 18.63%, which is the best efficiency among the DA‐based OSCs, higher than the other two systems (16.53% for PM6:DY‐V and 17.25% for PM6:DY2F‐V). The synergy of fluorination and vinylene‐linkage in DYF‐V‐based devices enhances intra/intermolecular interactions, leading to stronger crystallinity, improved phase segregation, reduced energy disorder, and robust toughness. The transient absorption technique suggests a faster exciton dissociation and reduced extent of recombination are realized in the PM6:DYF‐V, therefore leading to a long‐lived carrier lifetime for high efficiencies. Overall, proper fluorination on GDAs with rigid coplanar backbones paves one way for broader photon harvesting, stronger packing, and robust stability in high‐performance OSCs.

## 2 Results and Discussion

**Figure 1:**
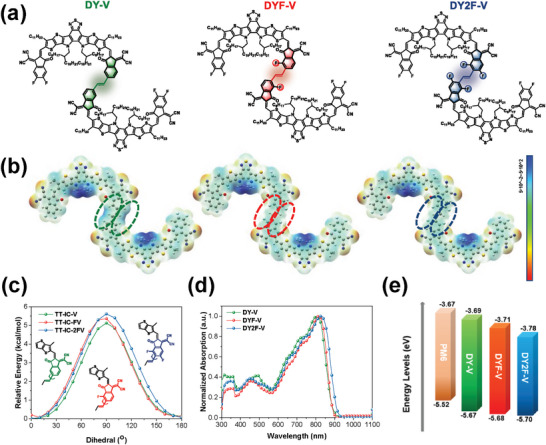
a) Chemical structures of DY‐V, DYF‐V and DY2F‐V. b) ESP distributions of DY‐V, DYF‐V, and DY2F‐V calculated by a DFT method at the B3LYP/6‐31G(d,p) set. c) Potential energy surface scan of model compounds TT‐IC‐V, TT‐IC‐FV, and TT‐IC‐2FV through the above DFT method. d) Normalized UV–Vis absorption spectra of neat films. e) Energy levels of PM6, DY‐V, DYF‐V, and DY2F‐V.

The chemical structures of DY‐V, DYF‐V, and DY2F‐V are depicted in **Figure** **1**a. The synthesis routes of the two GDAs with fluorination and vinylene linkages are similar to previous report,^[^ ^10^, ^16^, ^19^ ^]^ whose synthesis details and characterizations data, including ^1^H and ^13^C NMR spectra and mass spectra (Figures S2–S11, Supporting Information), can be found in the Supporting Information. Additionally, glass transition temperatures (*T*_g_s, Figure S12, Supporting Information; **Table** **1**) of the GDAs are precisely correlated to their thermal stability. DYF‐V and DY2F‐V exhibit *T*_g_s of 158 and 156 °C, slightly higher than that of DY‐V (*T*_g_ = 152 °C), indicating denser condensed states are formed in both fluorinated GDAs with higher thermal stability as we proposed.

**Table 1:** The thermal, optical, and electrochemical properties of DY‐V, DYF‐V, and DY2F‐V.

Materials	*T*_g_ [°C]	*λ*_max,sol_ [nm]	*λ*_max,film_ [nm]	*λ*_onset,film_ [nm]	*E*_g,opt_ [eV]	HOMO [eV]	LUMO [eV]
DY‐V	152	770	807	884	1.40	−5.67	−3.69
DYF‐V	158	779	810	889	1.39	−5.68	−3.71
DY2F‐V	156	801	827	907	1.37	−5.70	−3.78

Density functional theory (DFT) calculations are performed using the B3LYP/6‐31G (d,p) basis set to reveal different fluorinated impacts on molecular conformations of the GDAs (Figure S13a, Supporting Information). Due to the vinylene‐linkage strategy, both DY‐V and DYF‐V possess better coplanar backbones with broad electron cloud distributions between the two monomer units, while the DY2F‐V suggests a slightly twisting backbone due to the repulsion of fluorine atoms, indicative of weaker intramolecular conjugation. The electrostatic potentials (ESP) results are shown in Figure 1b, positive charges concentrate at the blue region while negative charges at the red region. As the fluorine number increases at the linking end group moieties, the ESP becomes more negative from DY‐V to DYF‐V and DY2F‐V, beneficial for the charge separation and electron delocalization in photovoltaic processes.^[^ ^17^ ^]^ Furthermore, potential energy surface scanning of representative compounds, including TT‐IC‐V, TT‐IC‐FV, and TT‐IC‐2FV indicates that the torsional potential improves from DY‐V to DYF‐V and DY2F‐V (Figure 1c).^[^ ^18^ ^]^ The introduction of fluorine atoms enhances the torsion‐free rigidity of the GDAs, owing to the conformational locking between F…S and F…H non‐covalent interactions.^[^ ^14f^ ^]^ Overall, DFT calculations demonstrate that DYF‐V with partial fluorination exhibits minimized intramolecular twisting, which will promote intermolecular packing and charge transport in devices. Nonetheless, whether multiple fluorine leads to better device performance needs to be further explored in the following study.

Subsequently, the UV–vis spectra of three GDAs in the thin‐film state were recorded in Figure 1d and Table 1. DY‐V displays a maximum absorption peak (*λ*_max,film_) at 807 nm, while the *λ*_max,film_s of DYF‐V and DY2F‐V exhibit bathochromic *λ*_max,film_s at 810 and 827 nm, respectively, attributed to the stronger ICT effect of the additional fluorination. In the solution state (Figure S14, Supporting Information), the absorption peaks (*λ*_max,sol_s) of the GDAs also display a similar trend as above. In films, DYF‐V and DY2F‐V exhibit smaller optical bandgaps (*E*_g,opt_s) of 1.97 and 1.92 eV, calculated by the red‐shifted absorption onsets of 889 and 907 nm, respectively, in comparison with DY‐V (1.98 eV and 884 nm). Therefore, the fluorination strategy effectively extends the light‐harvesting range of GDAs. To investigate the electrochemical properties of the GDAs, cyclic voltammetry (CV) was employed to assess their energy levels (Figure S15, Supporting Information). As shown in Figure 1e, the lowest unoccupied molecular orbital (LUMO) and the highest occupied molecular orbital (HOMO) levels of DY2F‐V were calculated as −3.78/‐5.70 eV, which are lower than DYF‐V (−3.71/−5.68 eV) and DY‐V (−3.69/−5.67 eV). This can be attributed to the electron‐withdrawing effect of fluorine in DYF‐V and DY2F‐V. Meanwhile, the calculated frontier molecular orbitals and bandgaps (Figure S13b, Supporting Information) agree well with the experimental trend, ensuring our rational synergistic design strategy.

**Figure 2:**
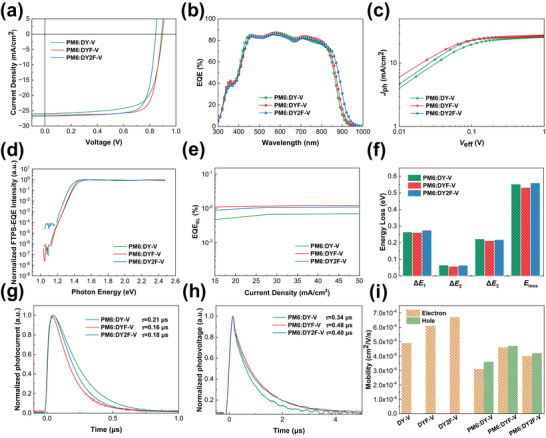
a) *J‐V* characteristics. b) EQE spectra. c) *J*_ph_–*V*_eff_ curves. d) Normalized EQE in the low‐energy region measured by Fourier transform photocurrent spectroscopy (FTPS). e) EL quantum efficiency (EL_EQE_) as dependent on current density. f) *E*_loss_ and its detailed three parts of Δ*E*_1_, Δ*E*_2_, and Δ*E*_3_ values. g) TPC and h) TPV measurements. i) Carrier mobility results of the pristine and blend devices.

To explore the synergistic effect of these GDAs on OSCs, devices were fabricated with a conventional structure of ITO/PEDOT:PSS/active layer/PNDIT‐F3N/Ag. PM6 was used for its complementary absorption and suitable energy level alignment with the dimeric acceptors in this work and was purchased from Volt‐Amp Optoelectronics Tech. Co., Ltd, Dongguan, China. The corresponding current density‐voltage (*J*–*V*) characteristics are shown in **Figure** **2**a. As shown in **Table** **2**, a PCE of 18.63% can be obtained from the PM6:DYF‐V‐based devices, which is the highest efficiency among the DA‐based OSCs. The remarkable performance mainly resulted from the higher short‐circuit current (*J*_SC_) of 26.62 mA cm^−2^ and significantly increased fill factor (FF) of 78.56%, thus outperforming the non‐fluorinated PM6:DY‐V (16.53%). Despite the multi‐fluorination degree of DY2F‐V with a higher *J*_SC_ of 26.83 mA cm^−2^ in devices, the relatively low open‐circuit voltage (*V*_OC_) of 0.84 V and FF of 76.17% lead to an inferior efficiency of 17.25%, which might be due to the slightly higher extent of energy disorder in blends. To explain the different *J*_SC_s, the external quantum efficiency (EQE) spectra of these devices were characterized. The EQE response of the PM6:DYF‐V can approach 80–85% in the range of 440–730 nm (Figure 2b). Besides, PM6:DYF‐V and PM6:DY2F‐V suggest wider EQE ranges, which contribute to the *J*_SC_s of the device as discussed. The integrated *J*_SC_s calculated from the corresponding EQE spectra are 25.35, 25.68, and 26.02 mA cm^−2^ for the DY‐V, DYF‐V, and DY2F‐V‐based OSCs, respectively, consistent well with the values obtained from the *J–V* curves.

**Table 2:** The optimal photovoltaic parameters of PM6:DY‐V, PM6:DYF‐V, and PM6:DY2F‐V‐based devices under AM 1.5G Illumination (100 mW cm^−2^).

Active layer	*V*_OC_ [V]	*J*_SC_ [mA cm^−2^]	*J*_SC,cal_ [mA cm^−2^]^a)^	FF [%]	PCE [%]
PM6:DY‐V	0.90	26.04	25.35	70.77	16.53
PM6:DYF‐V	0.89	26.62	25.68	78.56	18.63
PM6:DY2F‐V	0.84	26.83	26.02	76.17	17.25

^a)^ Calculated current density from the corresponding EQE curves.

Photoluminescence (PL) quenching experiments were measured to understand the difference in their EQE spectra, which were excited at 785 nm and shown in Figure S16 (Supporting Information). The PL quenching efficiency of PM6:DY‐V blend is only 85%, while PM6:DYF‐V and PM6:DY2F‐V blends suggest higher values of 90% and 91%, respectively. The introduction of fluorine atoms produces a higher extent of hole transfer in the exciton dissociation process and well explains the higher EQE response of DYF‐V‐ and DY2F‐V‐based devices. To further learn exciton dissociation in the three devices, the charge dissociation possibilities (P(E,T) = (*J*_ph_/*J*_sat_)) of DY‐V, DYF‐V, and DY2F‐V‐based devices were investigated (Figure 2c; Table S2, Supporting Information), which demonstrated the similar trends, where the DYF‐V‐based OSC exhibited the highest exciton dissociation efficiency.

Moreover, we investigated the origin of energy loss (*E*_loss_) of three DA‐based devices (Figure 2d–f; Table S3, Supporting Information). Normalized electroluminescence (EL) and highly sensitive EQE spectra of the three devices were processed for accurate bandgap calculation (*E*_g_, Figure S17, Supporting Information). PM6:DYF‐V and PM6:DY2F‐V presented smaller *E*_g_s of 1.42 and 1.40 eV, compared to that of PM6:DY‐V (1.45 eV), which leads to a trade‐off between the radiative recombination (Δ*E*_2_) and non‐radiative recombination (Δ*E*_3_).^[^ ^14f^ ^]^ Overall, the total *E*_loss_s were determined to be 0.55, 0.53, and 0.56 eV for PM6:DY‐V, PM6:DYF‐V, and PM6:DY2F‐V, respectively. One plausible reason for the larger *E*_loss_ in DY2F‐V is that the excess F may cause the steric effect or electrostatic repulsion in the packing state, thus a relatively high energy disorder. The smaller *E*_loss_ of DYF‐V‐based devices can be attributed to the regular fluorine‐induced conformational locking in PM6:DYF‐V, therefore both suppressed vibrational state and non‐radiative recombination.

The charge extraction and recombination process were further examined using transient photocurrent (TPC) and transient photovoltage (TPV) techniques. The TPC decay times for PM6:DY‐V, PM6:DYF‐V, and PM6:DY2F‐V were found to be 0.21, 0.16, and 0.18 µs, respectively, as shown in Figure 2g. The PM6:DYF‐V and PM6:DY2F‐V‐based devices sweep out charge faster, implying a better charge extraction capacity. TPV measurements (Figure 2h) reveal that DYF‐V‐based OSCs have the lowest non‐geminate charge recombination with the longest carrier lifetime of 0.48 µs relative to the PM6:DY‐V (0.34 µs) and PM6:DY2F‐V (0.40 µs). Light‐intensity‐dependent experiments (Figure S18, Supporting Information) on *V*_OC_ and *J*_SC_ further support these findings, where the DYF‐V‐based OSC exhibits the lowest extent of bimolecular and trap‐assisted charge recombination, resulting in a higher FF value.

In terms of charge transport properties of these systems, space‐charge‐limited current (SCLC) measurements (Figure S19, Supporting Information) were carried out, with device structures of ITO/PEDOT:PSS/PM6: DA/MoO_3_/Ag and ITO/ZnO/PM6:DA/PNDIT‐F3N/Ag, respectively, for the hole‐ and electron‐only diodes. As shown in Figure 2i; Table S4 (Supporting Information). The hole/electron mobilities (*µ*_h_/*µ*_e_) are 3.6/3.1 × 10^−4^, 4.7/4.6 × 10^−4^ and 4.2/4.0 × 10^−4^ cm^2^ V^−1^ s^−1^ for PM6:DY‐V, PM6:DYF‐V and PM6:DY2F‐V, respectively. The more balanced and higher charge mobilities could be found in DYF‐V‐ and DY2F‐V‐based devices than in DY‐V‐based devices. As for the e‐only device for pristine GDAs, both fluorinated DYF‐V and DY2F‐V suggest higher *µ*_e_s of 6.1 × 10^−4^ and 6.7 × 10^−4^ cm^2^ V^−1^ s^−1^ than DY‐V (4.9 × 10^−4^ cm^2^ V^−1^ s^−1^). The increased mobility is due to the introduction of fluorine atoms that increased both intra/intermolecular interactions, leading to rapid charge transport and a more balanced *µ*_h_/*µ*_e_ for decent FFs in PM6:DYF‐V devices.

**Figure 3:**
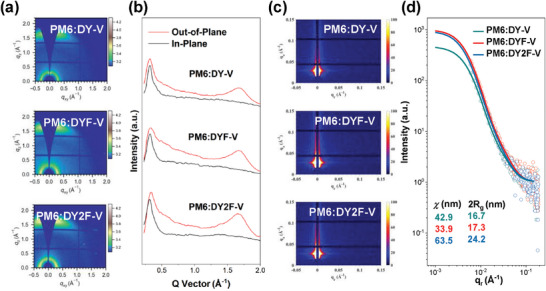
a) 2D GIWAXS patterns of PM6:DY‐V, PM6:DYF‐V, and PM6:DY2F‐V blend films and b) the corresponding 1D GIWAXS line‐cuts of the in‐plane (black line) and out‐of‐plane (red line) directions. c) GISAXS 2D diffraction patterns of DY‐V, DYF‐V, and DY2F‐V‐based blend films and d) the corresponding GISAXS intensity profiles and best fittings along the in‐plane directions.

To correlate the carrier mobility with morphological properties, grazing incidence wide‐angle X‐ray diffraction (GIWAXS) experiments were conducted to investigate the blend and pristine films. As shown in **Figure** **3**a,b and Table S5 (Supporting Information), all three blends exhibited a predominant face‐on orientation with diffraction peaks located at 1.69, 1.67 and 1.66 Å^−1^, respectively, corresponding to the π–π stacking distances of 3.71, 3.69 and 3.68 Å for PM6:DY‐V, PM6:DYF‐V and PM6:DY2F‐V, respectively, demonstrating a denser packing mode as fluorination increased. Besides, the coherence lengths (CCLs) of PM6:DYF‐V and PM6:DY2F‐V blends also exhibit larger values of 28.69 and 29.75 Å than PM6:DY‐V (25.69 Å), indicative of a stronger crystallinity in the PM6:DYF‐V and PM6:DY2F‐V. As for the pristine films in Figure S20 (Supporting Information), both DYF‐V and DY2F‐V showed a smaller π–π stacking distance of 3.71 Å, compared to the DY‐V (3.72 Å), suggesting a denser packing in the two fluorinated GDAs. Despite a slightly distorted conformation in DY2F‐V, it still exhibits denser packing, likely due to the stronger interaction by multiple fluorinations. Therefore, fluorination effectively improves the intra/intermolecular interaction of the GDAs, resulting in enhanced crystallinity for faster charge transport and increased FFs in devices as above. To figure out why the DYF‐V with a moderate fluorination degree provided a better performance, grazing‐incidence small‐angle X‐ray scattering (GISAXS) experiments were conducted to study the phase segregation of the three blends. The 2D patterns are shown in Figure 3c, while 3d presents the in‐plane direction intensity profiles of the three blends, and the best fittings by a fractal‐like network model. As the fluorination degree increased, the average crystalline domain sizes (2R_g_) of the DA phase are calculated to be 16.7, 17.3, and 24.2 nm for PM6:DY‐V, PM6:DYF‐V, and PM6:DY2F‐V, respectively, which is consistent with the GIWAXS results. However, the intermixing amorphous domain spacings (*χ*) are 42.9, 33.9, and 63.5 nm for the DY‐V, DYF‐V, and DY2F‐V‐based blends, respectively. PM6:DYF‐V demonstrates an increasing crystalline phase ratio and a reduced amorphous mixing phase length, implying a higher domain purity for superior phase segregation and the best FF value. As for the DY2F‐V‐based blend, the major reason for the relatively low device performance is due to the poor domain purity, which limits its charge transport and increases recombination in devices. Therefore, tailoring aggregation through proper fluorination for ideal phase segregation is the key to realizing a rapid photovoltaic process. Furthermore, the surface morphology of the three blends is characterized by atomic force microscopy (AFM, Figure S21, Supporting Information). The height and phase images reveal that the DYF‐V‐based blend forms a relatively smooth and uniform surface morphology with a smaller root‐mean‐square (RMS) than the other two, enabling the charge extraction process as above discussed.

**Figure 4:**
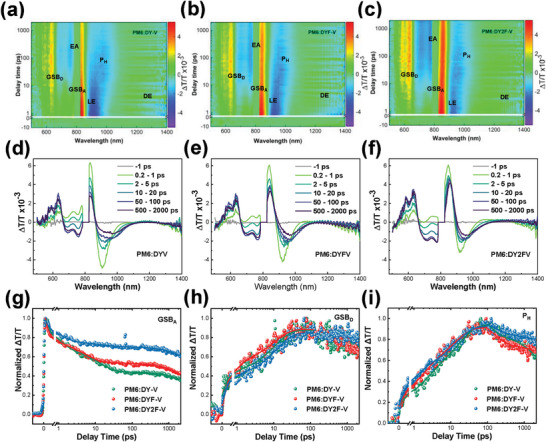
Ultrafast transient absorption spectroscopy of the samples. a–c) 2D TA contour plots of dimer‐V blends under acceptor excitation (800 nm) of PM6: DY‐V, PM6: DYF‐V and PM6: DY2F‐V. d–f) Transient absorption spectra of samples at different delay times after 800‐nm photoexcitation. g) GSB_D_ and GBS_A_ denote ground‐state bleaching from donor and acceptor, LE and DE stand for local exciton and delocalized state, EA is electro‐absorption band, and PH is a hole polaron. TA kinetics probed at maximum of acceptor and donor h) GSBs, and hole polaron i).

Transient absorption spectroscopy was carried out under excitation into the acceptor band (800 nm) with an average pulse energy of 2 µJ cm^−2^ (**Figure** **4**a–c; Figure S23, Supporting Information). After photoexcitation we observe an immediate, instrumental response‐limited rise of positive Δ*T*/*T* signal at 800–850 nm range accompanied by excited‐state absorption negative Δ*T*/*T* signal ≈900 nm, which both corresponds to local exciton (LE) formation. Together with that, another broad ESA band emerges beyond 1300 nm. According to previous reports, this band was attributed to the delocalized state (DE), appearing over different acceptor molecules at the same moiety,^[^ ^19^ ^]^ which is also observed in neat acceptor films. The delocalization of electron wavefunction with DE state reduces the interfacial Coulomb attraction of the electron‐hole pair promoting separation of local excitons into free carriers.^[^ ^20^ ^]^ At later times after photoexcitation, we also observe the formation of a positive Δ*T*/*T* signal within the absorption band of the donor (600–650 nm) due to hole transfer from the acceptor. The hole transfer leads to hole polaron (P_H_) formation and results in the appearance of another ESA band next to an LE one. Finally, the charge separation at the D/A heterojunction is evident from the appearance of the transient electro‐absorption band (EA) ≈750 nm arising from a Stark shift of the absorption spectrum by the local electric fields generated between electron–hole pairs during the separation process.^[^ ^21^ ^]^ Note that EA and P_H_ signals are absent in neat acceptor films (Figure S23, Supporting Information).

The dynamics of the abovementioned process are reflected in the TA kinetics probed at different spectral ranges (Figure 4g–i; Table S6, Supporting Information). From the fastest initial quench of GSB_A_, we conclude that the DYF‐V sample promotes the most efficient formation of the DE state. From the biexponential fit of GSB_D_, we can see that PM6:DY2F‐V demonstrates the slowest exciton diffusion in the bulk acceptor domains (τ_2_ component in Table S1, Supporting Information) due to larger domain size, being consistent with TPC/TPV and GIWAXS data. At the same time, the exciton dissociation near the donor/acceptor interface (τ_1_ component in Table S6, Supporting Information) is slightly longer for PM6:DYF‐V, which may be related to a longer carrier lifetime in this sample. Nevertheless, the same order of fast and slow time components of GSB_D_ turns us to the conclusion that fluorination does not significantly alter the hole transfer rate. However, we note improvements in polaron kinetics for fluorinated samples with the fastest build‐up time for PM6:DY2FV, being consistent with the fastest exciton dissociation at the donor/acceptor interface for this sample.

The most distinct effect of fluorination is observed in the EA band (700–750 nm). The fluorinated samples exhibit a much stronger EA band with longer build‐up times (Figure S24, Supporting Information). Compared to the DY‐V sample, requiring ≈75 ps to build up, long‐range charge separation in fluorinated samples occurs at a much slower rate reaching its maxima within 100 and 190 ps for DYF‐V and DY2F‐V samples, correspondingly. That indicates the decreasing of energy offset between donor and acceptor after fluorination being consistent with results obtained by CV measurements and DFT calculations. Also, longer building‐up times for the EA band may be related to more efficient diffusion of excitons to the donor/acceptor interface due to better morphology of donor and acceptor domains. In this regard, we conclude that the main effect on the device performance comes from more efficient charge separation in the single‐fluorinated sample and its most balanced charge dissociation and recombination rates, as well as morphological causes such as better phase separation, as revealed by the GISAXS analysis.

Thermal stability tests at 80 °C yielded similar results, where the PM6:DYF‐V maintains the highest level of stability (Figure S22a, Supporting Information). The light stability of DA‐based OSCs was evaluated by monitoring their PCE under continuous 1‐sun illumination (Figure S22b, Supporting Information). The *t*_80%_ lifetimes, which represent the time taken for the PCE to degrade to 80%, were found to follow the order of PM6:DY‐V < PM6:DY2F‐V < PM6:DYF‐V. The PM6:DYF‐V exhibited a high PCE (>18%) and demonstrated outstanding stability, with a *t*_80%_ lifetime exceeding 700 h. The superior stability of DYF‐V‐based devices was due to the stable conformation and denser morphology property, achieved by the synergy of vinylene linkage and precise fluorination as discussed.

## 3 Conclusion

The study explores the synergy of fluorination and coplanar linkage of GDAs by connecting one vinylene linker with the mono‐/di‐fluorinated end group for efficient and stable OSCs. Through precise fluorination, DYF‐V with a moderate fluorination degree demonstrates a stronger ICT effect and torsion‐free backbones with red‐shifted absorption and denser packing modes. The OSC based on PM6:DYF‐V exhibits a higher PCE of 18.6% compared to its counterpart, PM6:DY2F‐V (17.3%) and PM6:DY‐V (16.5%). Theoretical and morphological analyses indicate that the fluorination of DYF‐V‐based devices enhances intra/intermolecular interactions, leading to improved crystallinity, superior phase segregation, and reduced energy disorder, therefore higher carrier mobility and suppressed energy loss. Throughout transient techniques, DYF‐V‐based blends also suggest faster exciton dissociation, and suppressed charge recombination. Besides, beneficial from the lower occupied energy level, the DYF‐V‐based devices deliver robust toughness and stability. Our work demonstrates that fluorination of GDAs with rigid coplanar backbones is an effective strategy for extending photon harvesting and strengthening molecular packing for efficient and stable OSCs.

## Conflict of Interest

The authors declare no conflict of interest.

## Supporting information



Supporting Information

## Data Availability

The data that support the findings of this study are available from the corresponding author upon reasonable request.
