# Supplementary material for: Coplanar Dimeric Acceptors with Bathochromic Absorption and Torsion‐Free Backbones through Precise Fluorination Enabling Efficient Organic Photovoltaics with 18.63% Efficiency
Source: Adv Sci (Weinh). 2025 Jan 20;12(10):2410826. doi: 10.1002/advs.202410826 (PMC11904988; doi:10.1002/advs.202410826)
Supplement: Supplementary file 1 — Supporting Information [file ADVS-12-2410826-s001.pdf]

ADVANCED SCIENCE
Open Access

Supporting Information

for *Adv. Sci.*, DOI 10.1002/advs.202410826

Coplanar Dimeric Acceptors with Bathochromic Absorption and Torsion-Free Backbones through Precise Fluorination Enabling Efficient Organic Photovoltaics with 18.63% Efficiency

Wei Liu, Weiwei Wu, Aleksandr A. Sergeev, Jia Yao, Yuang Fu, Chung Hang Kwok, Ho Ming Ng, Chunliang Li, Xiaojun Li, Sai Ho Pun, Huawei Hu, Xinhui Lu, Kam Sing Wong, Yongfang Li, He Yan and Han Yu**

Coplanar Dimeric Acceptors with Bathochromic Absorption and Torsion-Free Backbones through Precise Fluorination Enabling Efficient Organic Photovoltaics with 18.63% Efficiency

Wei Liu, Weiwei Wu, Aleksandr A. Sergeev, Jia Yao, Yuang Fu, Chung Hang Kwok, Ho Ming Ng, Chunliang Li, Xiaojun Li, Sai Ho Pun, Huawei Hu, Xinhui Lu, Kam Sing Wong, Yongfang Li, He Yan, Han Yu**

W. Liu, H. Yan, H. Yu
Guangdong-Hong Kong Joint Laboratory for Carbon Neutrality, Jiangmen Laboratory of Carbon Science and Technology, Jiangmen, Guangdong 529199 (China)
E-mail: hyan@ust.hk; hyuak@connect.ust.hk

W. Liu, W. Wu, J. Yao, H. M. Ng, C. H. Kwok, C. Li, L. Zhou, S. H. Pun, H. Yan, H. Yu
Department of Chemistry
Hong Kong Branch of Chinese National Engineering Research Center for Tissue Restoration and Reconstruction, Hong Kong University of Science and Technology
Clear Water Bay, Kowloon, Hong Kong 999077, (China)

A. A. Sergeev, K. S. Wong
Department of Physics
William Mong Institute of Nano Science and Technology, The Hong Kong University of Science and Technology
Clear Water Bay, Kowloon, Hong Kong 999077, (China)

Y. Fu, X. Lu
Department of Physics
Chinese University of Hong Kong
999077 New Territories, Hong Kong (China)

H. Hu
State Key Laboratory for Modification of Chemical Fibers and Polymer Materials
College of Materials Science and Engineering
Donghua University, Shanghai 201620 (China)
X. Li, Y. Li
Beijing National Laboratory for Molecular Sciences, CAS Key Laboratory of Organic Solids, Institute of Chemistry, Chinese Academy of Sciences
Beijing,100190, (China)

H. Yan, H. Yu
Hong Kong University of Science and Technology-Shenzhen Research Institute, No. 9, Yuexing 1st RD, Hi-tech Park, Nanshan, Shenzhen 518057, (China)

H. Yan
Hong Kong University of Science and Technology Fok Ying Tung Research Institute
S&T Building, Nansha IT Park, Guangzhou City, 511458, P. R. (China)

H. Yu
Department of Applied Biology and Chemical Technology, The Hong Kong Polytechnic University, Hung Hom, Kowloon, Hong Kong 999077, (China)

Supporting Information

Table of Contents

Materials and Methods ……………………………………………………………………..**S3**
Material Synthesis………………………………………………………………….……….**S8**
Supplementary Figures…………………………………………………………………….**S13**
Supplementary Tables ………………………………………………………………… **S34**

Materials and Methods.

General Information. ^1H and ^{13}C NMR spectra were recorded on a Bruker AV-400 MHz NMR spectrometer. Chemical shifts are reported in parts per million (ppm, δ). ^1H NMR and ^{13}C NMR spectra were referenced to tetramethylsilane (0 ppm) for $CDCl_3$. Mass spectra were collected on a MALDI Micro MX mass spectrometer, or an API QSTAR XL System. Compound 1 and 2-butyloctyl iodide were prepared according to literature procedures.

Materials. PM6 (M_n=24.2 kDa) was purchased from Volt-Amp Optoelectronics Tech. Co., Ltd, Dongguan, China. Tetrahydrofuran was freshly distilled before use from sodium using benzophenone as the indicator. All other reagents and chemicals were purchased from commercial sources and used without further purification.

Estimation of glass-transition temperature. UV–vis spectroscopy was used to determine the glass transition temperature (T_g). The absorption spectra of DY-V, DYF-V and DY2F-V films were measured with increasing temperatures from 20 to 250 °C. For the preparation of DY-V, DYF-V and DY2F-V films, we used the same processing conditions (i.e., solvent, concentration, and spin-coating speed) as those for the OSC fabrication, to precisely correlate the estimated T_gs with those in the OSC device. Then, the deviation metric (DMT) of each absorption spectra was calculated, following the method reported by Samuel E. Root et al.

$$\mathrm{DM_T} \equiv \sum_{\lambda_{\min}}^{\lambda_{\max}} [\mathrm{I_{RT}}(\lambda) - \mathrm{I_T}(\lambda)]^2$$

where λ is the wavelength, λ_{max} and λ_{min} are the upper and lower bounds of the optical sweep, respectively, IRT(λ) and IT(λ) are the normalized absorption intensities of the as-cast (room temperature) and annealed films, respectively. Then, the T_g is determined to be the point where the two interpolated lines in low- and high-temperature regions intersect.

DFT calculation. Geometries of the repeating units and relaxed potential energy scan of three model compounds in neutral state as calculated by B3LYP/6-31g (d, p).

Optical characterizations. Film UV-V is absorption spectra were acquired on a Perkin Elmer Lambda 20 UV/VIS Spectrophotometer. All film samples were spin-cast on ITO substrates. UV-V is absorption spectra were collected from the solution of three small molecules with the concentration of 1.0 × 10^{-5} M in chloroform. A cuvette with a stopper (Sigma Z600628) was used to avoid volatilization during the measurement.

Electrochemical characterizations. Cyclic voltammetry was carried out on a CHI610E electrochemical workstation with three electrodes configuration, using Ag/AgCl as the reference electrode, a Pt plate as the counter electrode, and a glassy carbon as the working electrode. 0.1 mol L^{-1} tetrabutylammonium hexafluorophosphate in anhydrous acetonitrile was used as the supporting electrolyte. The polymer and small molecules were drop-cast

onto the glassy carbon electrode from chloroform solutions (5 mg/mL) to form thin films. Potentials were referenced to the ferrocenium/ferrocene couple by using ferrocene as external standards in acetonitrile solutions. The scan rate is 100 mV s^{-1}.

AFM analysis. AFM measurements were performed by using a Scanning Probe Microscope Dimension 3100 in tapping mode. All film samples were spin-cast on ITO substrates.

Solar cell fabrication and testing. OSCs were made with a device structure of ITO/PEDOT: PSS/PM6: DA/PNDIT-F3N/Ag. The patterned ITO-coated glass was scrubbed by detergent and then cleaned inside an ultrasonic bath by using deionized water, acetone, and isopropyl alcohol sequentially and dried overnight in an even. Before use, the glass substrates were treated in a UV-Ozone Cleaner for 20 min to improve its work function and clearance. A thin PEDOT: PSS layer with a thickness of about 40 nm was spin-coat onto the ITO substrates at 4500rmp for 40 s, and then dried at 150 °C for 15 min in air. The PEDOT: PSS coated ITO substrates were transferred to a N_2-filled glove box for further processing. The donor: acceptor blends with weight ratio of 1:1 and total concentration of 16 mg/mL dissolved in chloroform with 1% CN as additive. The blend solution was spin-cast on the top of PEDOT: PSS layer immediately after being stirred on a hotplate of 65 °C for 30 minutes at 2500 rpm for 40 s. Then it was annealed at 100 °C for 5 min to remove the solvent. A thin layer of PNDIT-F3N (~10 nm) was cast onto processed active layer, and Ag layer (~100 nm) was deposited in thermal evaporator under vacuum of 5×10^{-5} Pa through a shadow mask. The optimal blend thickness measured on a Bruker Dektak XT stylus profilometer was about 100nm. The current-voltage(J-V) characteristic curves of all packaged devices were measured by using a Keithley 2400 Source Meter in air. Photocurrent was measured under AM 1.5G (100 mW cm^{-2}) using a Newport solar simulator in an Air. The light intensity was calibrated using a standard Si diode (with KG5 filter, purchased from PV Measurement) to bring spectral mismatch to unity. EQEs were measured using an Enlitech QE-S EQE system equipped with a standard Si diode. Monochromatic light was generated from a Newport 300W lamp source.

EQE measurements. EQEs were measured using an Enlitech QE-S EQE system equipped with a standard Si diode. Monochromatic light was generated from a Newport 300W lamp source.

TPC measurements. Transient photocurrent (TPC) measurements: Relevant control and MSM solar cells were excited with a 405 nm laser diode. The transient photocurrent response of the devices at short circuit condition to a 200 μs square pulse from the LED with no background illumination. The current traces were recorded on a Tektronix DPO3034 digital oscilloscope by measuring the voltage drop over a 5-ohm sensor resistor in series with the solar cell. DC voltage was applied to the solar cell with an MRF544 bipolar junction transistor in a common collector amplifier configuration.

TPV measurements. Transient photovoltage (TPV) measurements: In the TPV

measurements, a 405 nm laser diode was used to keep the organic solar cells in the V_{OC} conditions. Measuring the light intensity with a highly linear photodiode and driving the laser intensity with a waveform generator (Agilent 33500B) allowed reproducible adjustments of the light intensities on 1 sun. Moreover, a small perturbation was induced with a second 405 nm laser diode. The intensity of the short laser pulse was adjusted to keep the voltage perturbation below 10 mV. After the pulse, the voltage decays back to its steady state value in a single exponential decay.

Hole-mobility measurements. The hole-mobilities were measured using the space charge limited current (SCLC) method, employing a device architecture of ITO/PEDOT:PSS/blend film/MoO_3/Al. The mobilities were obtained by taking current-voltage curves and fitting the results to a space charge limited form, where the SCLC is described by:

$$J = \frac{9\epsilon_0 \epsilon_r \mu (V_{\mathrm{appl}} - V_{\mathrm{bi}} - V_{\mathrm{s}})^2}{8L^3}$$

Where ε_0 is the permittivity of free space, ε_r is the relative permittivity of the material (assumed to be 3), μ is the hole mobility and L is the thickness of the film. From the plots of $J^{1/2}$ vs $V_{\mathrm{appl}} - V_{\mathrm{bi}} - V_{\mathrm{s}}$, hole mobilities can be deduced.

Electron mobility measurements. The electron mobilities were measured using the SCLC method, employing a device architecture of ITO/ZnO/blend film/PNDIT-F3N/Al. The mobilities were obtained by taking current-voltage curves and fitting the results to a space charge limited form, where the SCLC is described by:

$$J = \frac{9\epsilon_0 \epsilon_r \mu (V_{\mathrm{appl}} - V_{\mathrm{bi}} - V_{\mathrm{s}})^2}{8L^3}$$

Where ε_0 is the permittivity of free space, ε_r is the relative permittivity of the material (assumed to be 3), μ is the hole mobility and L is the thickness of the film. From the plots of $J^{1/2}$ vs $V_{\mathrm{appl}} - V_{\mathrm{bi}} - V_{\mathrm{s}}$, electron mobilities can be deduced.

GIWAXS & GISAXS Characterization. The grazing incident wide-angle X-ray scattering (GIWAXS) and the grazing incident small-angle X-ray scattering (GISAXS) measurements were carried out with a Xeuss 2.0 SAXS/WAXS laboratory beamline using a Cu X-ray source (8.05 keV, 1.54 Å) and a Pilatus3R 300K detector. The incidence angle is 0.2°. The coherence length was calculated using the Scherrer equation: CL = 2πK/Δq, 3 Where Δq is the full-width at half-maximum of the peak and K is a shape factor (1 was used here).

FTPS-EQE measurements. Fourier-transform photocurrent spectroscopy external quantum efficiency (FTPS-EQE) spectra were measured by using a Vertex 70 from Bruker optics and QTH lamp. The EL signature was collected with a monochromator and detected with a Si-CCD detector.

Transient absorption spectroscopy. Measurements were performed using a custom-built

experimental setup with an amplified Ti:sapphire laser (Coherent Legend Elite), with pulse duration of 120 fs, centered at 800 nm and at a repetition rate of 1000 Hz. The pump pulses were generated using an optical parametric amplifier (Coherent Opera Solo) and then chopped to 500 Hz. The probe beam was traversed a mechanical translation stage, enabling a time delay of up to 2 ns between pump and probe pulses, and then was focused on an Yttrium Aluminium Garnet crystal plate. After passing through the photoexcited sample, the probe pulses were spectrally dispersed using grating and then collected with a silicon line CCD (Hamamatsu S8380) (visible components, 500-1000 nm range) or an InGaAs line CCD (Hamamatsu G11620) (IR components, 800-1600 nm range). The differential transmission signals at various delay times were calculated from the sequential probe shots corresponding to the pump on and off cases as $((T_{pump\ ON} - T_{pump\ OFF})/T_{pump\ OFF})$. Measurements were conducted under excitation in fundamental absorption band of the acceptor (800 nm) with an average flux of ~2 μJ cm^{-2}, which is close to 1-sun illumination.

Femtosecond transient absorption spectroscopy measurements. For the fsTA measurements, the pump wavelength was set to 800 nm to selectively excite the acceptor in the blend and the average pulse energy was 2 μJ cm^{-2}, corresponding to 1-sun illumination. After photoexcitation, we observe an immediate, instrumental response-limited rise of positive $\Delta T/T$ signal at the 800-850 nm range, corresponded to the ground-state bleaching of the acceptor (GSB^A) in Figure 4a, which is accompanied with excited-state absorption negative $\Delta T/T$ signal around 900 nm, which both corresponds to local exciton (LE) formation. Together with that, another broad excited-state absorption (ESA) band emerges beyond 1300 nm. According to previous reports [1], this band was attributed to the delocalized state (DE), appearing over different acceptor molecules at the same moiety. The delocalization of electron wavefunction with DE state reduces the interfacial Coulomb attraction of the electron-hole pair promoting separation of local excitons into free carriers[2]. At later times after photo-excitation we also observe the formation of a positive $\Delta T/T$ signal within absorption band of the donor (GSB_D) due to hole transfer from the acceptor. The hole transfer leads to hole polaron (PH) formation resulting in the appearance of another ESA band next to LE one. Finally, the charge separation at the D/A heterojunction is evident from the appearance of transient electro absorption band around 750 nm arising from a Stark shift of the absorption spectrum by the local electric fields generated between electron–hole pairs during the separation process [3].

Material Synthesis.

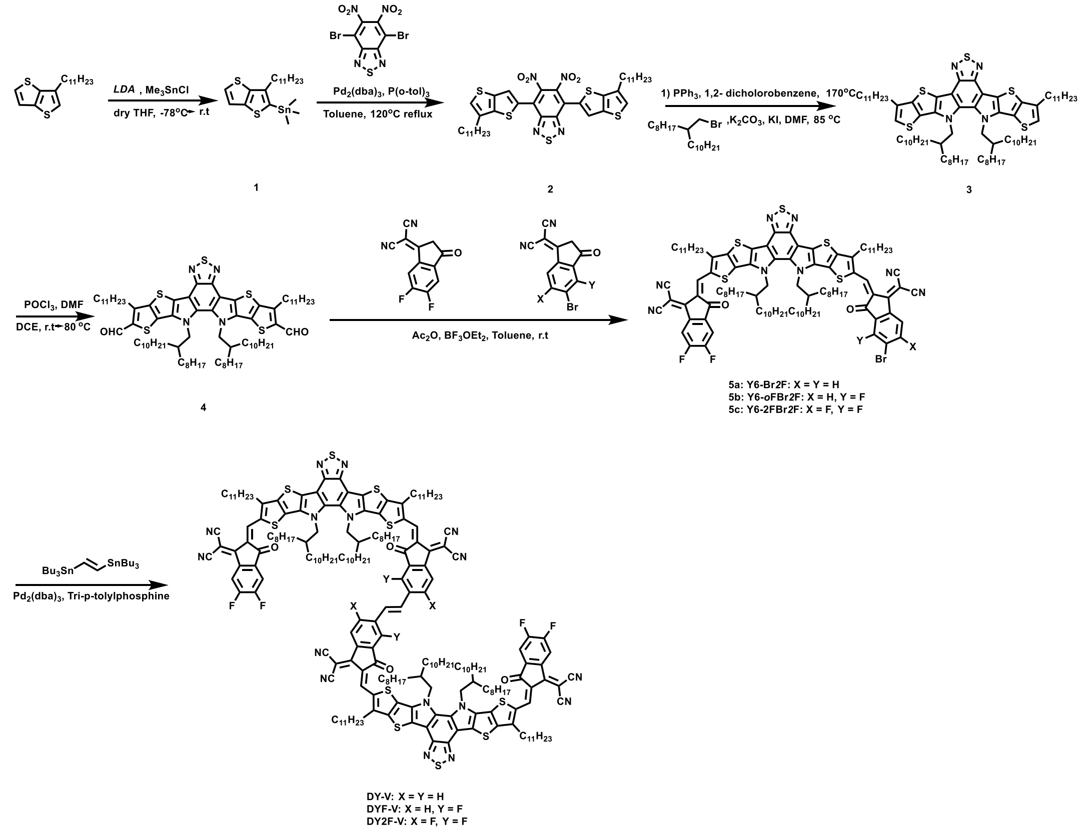

2-((Z)-2-((10-(((Z)-5-bromo-1-(dicyanomethylene)-4,6-difluoro-3-oxo-1,3-dihydro-2H-inden-2-ylidene)methyl)-12,13-bis(2-octyldodecyl)-3,9-diundecyl-12,13-dihydro-[1,2,5]thiadiazolo[3,4-e]thieno[2'',3'':4',5']thieno[2',3':4,5]pyrrolo[3,2-g]thieno [2',3':4,5]thieno[3,2-b]indol-2-yl)methylene)-5,6-difluoro-3-oxo-2,3-dihydro-1H-inden-1-ylidene)malononitrile 5b(Y6-*o*FBr2F).

Under the protection of argon, compound 4 (242 mg, 0.18 mmol) were added to a solvent mixture of chloroform (8 mL) and pyridine (0.5 mL). The reaction was placed in an oil bath at 65°C and stirred overnight. After the reaction, residual solvents were by removed at low pressure (< 300 mbar), and the was purified by flash silica gel chromatography with petroleum ether/DCM (v/v=2/1) as eluent to afford a crude product containing compound 5a (51 mg, yield 54%). ^{1}H NMR (CDCl$_3$, 400 MHz) δ 9.17 (d, *J* = 10.0 Hz, 2H), 8.57 (m, 1H), 8.41 (d, *J* = 8.4 Hz, 1H), 7.92 (m, 1H), 7.70 (m, 1H), 4.76 (d, *J* = 7.2 Hz, 4H), 3.23 (m, 4H), 2.12 (s, 2H), 1.87 (s, 4H), 1.86 (s, 4H), 1.12 (m, 120H). MS (MALDI-TOF) [M] calcd. for ($C_{106}H_{134}F_3N_8O_2S_5$):1849.49. Found: 1849.51.

2-((Z)-2-((10-(((Z)-5-bromo-1-(dicyanomethylene)-4,6-difluoro-3-oxo-1,3-dihydro-2H-inden-2-ylidene)methyl)-12,13-bis(2-octyldodecyl)-3,9-diundecyl-12,13-dihydro-

[1,2,5]thiadiazolo[3,4-e]thieno[2'',3'':4',5']thieno[2',3':4,5]pyrrolo[3,2-g]thieno[2',3':4,5]thieno[3,2-b]indol-2-yl)methylene)-5,6-difluoro-3-oxo-2,3-dihydro-1H-inden-1-ylidene)malononitrile 5c(Y6-2FBr2F) (45 mg, yield 78%) was prepared according to the same method as 5a. ^{1}H NMR ($CDCl_3$, 400 MHz) δ 9.12 (s, 2H), 8.53 (m, 1H), 8.30 (d, *J* = 8.0 Hz, 1H), 7.69 (d, J = 6.8 Hz, 1H), 4.78 (d, *J* = 6.8 Hz, 4H), 3.19 (d, *J* = 6.4 Hz, 4H), 2.15 (s, 2H), 1.86 (s, 4H), 1.13 (m, 184H). MS (MALDI-TOF) [M] calcd. for ($C_{106}H_{133}BrF_4N_8O_2S_5$):1867.48. Found: 1867.38.

2,2'-((2Z,2'Z)-(((((2Z,2'Z)-((E)-ethene-1,2-diyl)bis(1-(dicyanomethylene)-3-oxo-1,3-dihydro-2H-indene-5-yl-2-ylidene))bis(methanylylidene))bis(12,13-bis(2-octyldodecyl)-3,9-diundecyl-12,13-dihydro-[1,2,5]thiadiazolo[3,4-e]thieno [2'',3'':4',5']thieno[2',3':4,5]pyrrolo[3,2-g]thieno[2',3':4,5]thieno[3,2-b]indole-10,2-diyl))bis(methanylylidene))bis(5,6-difluoro-3-oxo-2,3-dihydro-1H-indene-2,1-diylidene))dimalononitrile (DYF-V). To a 25 mL Schlenk tube equipped with a stirring bar, trans-1,2-bis(tri-nbutylstannyl)ethylene (4.16 mg, 0.009 mmol) and dibrominated monomer 5b (30.0 mg, 0.016 mmol), $Pd_2(dba)_3$ (1.18 mg, 0.18×10^{-3} mmol), $P(o\text{-tolyl})_3$ (1.00 mg, 5.06×10^{-3} mmol), and toluene (2 mL) were added. The tube was purged with nitrogen and sealed under nitrogen flow and stirred at 110 ^{o}C for 3 days. Then the reaction mixture was cooled down to room temperature and precipitated into 200 mL of methanol. The solid residue was re-dissolved in chloroform (2 mL) and added slowly to methanol (20 mL). The precipitates were collected by filtration, washed with methanol, and dried in vacuum, leading to a deep blue solid as the product DYF-V (50 mg, yield 86%). ^{1}H NMR ($CDCl_3$, 400 MHz) δ 9.15 (d, *J* = 25.2 Hz, 4H), 8.59 (m, 4H), 8.06 (s, 2H), 7.67 (m, 4H), 4.83 (s, 8H), 3.18 (d, *J* = 49.2 Hz, 8H), 2.16 (s, 4H), 1.88 (s, 8H), 1.03 (m, 268H). ^{13}C NMR (101 MHz, CDCl3) *δ* 184.59, 181.13, 153.85, 146.99, 146.79, 146.07, 144.36, 136.96, 136.89, 136.80, 136.68, 134.61, 134.38, 132.61, 130.87, 128.81, 126.67, 119.37, 119.24, 118.64, 114.56, 114.19, 113.00, 112.12, 67.43, 31.30, 31.29, 31.17, 29.96, 29.76, 29.26, 29.11, 29.07, 29.02, 28.98, 28.94, 28.91, 28.82, 28.73, 28.71, 28.58, 24.80, 22.07. MS (MALDI-TOF) [M] calcd. for ($C_{214}H_{270}F_6N_{16}O_4S_{10}$):3565.21. Found: 3564.22.

2,2'-((2Z,2'Z)-(((((2Z,2'Z)-((E)-ethene-1,2-diyl)bis(1-(dicyanomethylene)-4,6-difluoro-3-oxo-1,3-dihydro-2H-indene-5-yl-2-ylidene))bis(methanylylidene))bis(12,13-bis(2-octyldodecyl)-3,9-diundecyl-12,13-dihydro-[1,2,5]thiadiazolo[3,4-e]thieno[2'',3'':4',5']thieno[2',3':4,5]pyrrolo[3,2-g]thieno[2',3':4,5]thieno[3,2-b]indole-10,2-diyl))bis(methanylylidene))bis(5,6-difluoro-3-oxo-2,3-dihydro-1H-indene-2,1-diylidene))dimalononitrile (DY2F-V) (50 mg, yield 86%) was prepared according to the same method as DYF-V. ^{1}H NMR ($CDCl_3$, 400 MHz) δ 9.16 (d, *J* = 6.0 Hz, 4H), 8.57 (m, 2H),

8.39 (d, J = 10.4 Hz, 2H), 7.77 (s, *J* = 7.6 Hz, 2H), 7.67 (m, 2H), 4.79 (d, *J* = 6.4 Hz, 8H), 3.23 (m, 8H), 2.15 (s, 4H), 1.88 (d, *J* = 7.2 Hz, 8H), 1.54 (s, 8H), 1.03 (m, 232H). ^{13}C NMR (101 MHz, $CDCl_3$) δ 153.76, 153.29, 146.88, 144.73, 144.64, 137.33, 137.16, 135.39, 133.48, 132.93, 132.72, 113.07, 31.30, 31.24, 31.17, 29.27, 29.15, 29.05, 29.02, 29.00, 28.95, 28.93, 28.93, 28.82, 28.77, 28.73, 28.59, 22.07, 22.00, 13.49. MS (MALDI-TOF) [M] calcd. for ($C_{214}H_{268}F_8N_{16}O_4S_{10}$):3601.19. Found: 3600.10.

2-((Z)-2-((10-(((Z)-5-bromo-1-(dicyanomethylene)-4,6-difluoro-3-oxo-1,3-dihydro-2H-inden-2-ylidene)methyl)-12,13-bis(2-octyldodecyl)-3,9-diundecyl-12,13-dihydro-[1,2,5]thiadiazolo[3,4-e]thieno[2'',3'':4',5']thieno[2',3':4,5]pyrrolo[3,2-g]thieno[2',3':4,5]thieno[3,2-b]indol-2-yl)methylene)-5,6-difluoro-3-oxo-2,3-dihydro-1H-inden-1-ylidene)malononitrile 5b(Y6-*o*FBr2F).

Under the protection of argon, compound 4 (242 mg, 0.18 mmol) were added to a solvent mixture of chloroform (8 mL) and pyridine (0.5 mL). The reaction was placed in an oil bath at 65°C and stirred overnight. After the reaction, residual solvents were by removed at low pressure (< 300 mbar), and the was purified by flash silica gel chromatography with petroleum ether/DCM (v/v=2/1) as eluent to afford a crude product containing compound 5a (51 mg, yield 54%). ^{1}H NMR ($CDCl_3$, 400 MHz) δ 9.17 (d, *J* = 10.0 Hz, 2H), 8.57 (m, 1H), 8.41 (d, *J* = 8.4 Hz, 1H), 7.92 (m, 1H), 7.70 (m, 1H), 4.76 (d, *J* = 7.2 Hz, 4H), 3.23 (m, 4H), 2.12 (s, 2H), 1.87 (s, 4H), 1.86 (s, 4H), 1.12 (m, 120H). MS (MALDI-TOF) [M] calcd. for ($C_{106}H_{134}F_3N_8O_2S_5$):1849.49. Found: 1849.51.

2-((Z)-2-((10-(((Z)-5-bromo-1-(dicyanomethylene)-4,6-difluoro-3-oxo-1,3-dihydro-2H-inden-2-ylidene)methyl)-12,13-bis(2-octyldodecyl)-3,9-diundecyl-12,13-dihydro-[1,2,5]thiadiazolo[3,4-e]thieno[2'',3'':4',5']thieno[2',3':4,5]pyrrolo[3,2-g]thieno[2',3':4,5]thieno[3,2-b]indol-2-yl)methylene)-5,6-difluoro-3-oxo-2,3-dihydro-1H-inden-1-ylidene)malononitrile 5c(Y6-2FBr2F) (45 mg, yield 78%) was prepared according to the same method as 5a. ^{1}H NMR ($CDCl_3$, 400 MHz) δ 9.12 (s, 2H), 8.53 (m, 1H), 8.30 (d, *J* = 8.0 Hz, 1H), 7.69 (d, J = 6.8 Hz, 1H), 4.78 (d, *J* = 6.8 Hz, 4H), 3.19 (d, *J* = 6.4 Hz, 4H), 2.15 (s, 2H), 1.86 (s, 4H), 1.13 (m, 184H). MS (MALDI-TOF) [M] calcd. for ($C_{106}H_{133}BrF_4N_8O_2S_5$):1867.48. Found: 1867.38.

2,2'-((2Z,2'Z)-(((((2Z,2'Z)-((E)-ethene-1,2-diyl)bis(1-(dicyanomethylene)-3-oxo-1,3-dihydro-2H-indene-5-yl-2-ylidene))bis(methanylylidene))bis(12,13-bis(2-octyldodecyl)-3,9-diundecyl-12,13-dihydro-[1,2,5]thiadiazolo[3,4-e]thieno[2'',3'':4',5']thieno[2',3':4,5]pyrrolo[3,2-g]thieno[2',3':4,5]thieno[3,2-b]indole-10,2-diyl))bis(methanylylidene))bis(5,6-difluoro-3-oxo-2,3-dihydro-1H-indene-2,1-diylidene))dimalononitrile (DYF-V). To a 25 mL Schlenk tube equipped with a stirring bar, trans-1,2-bis(tri-nbutylstannyl)ethylene (4.16 mg, 0.009 mmol) and dibrominated monomer 5b (30.0 mg, 0.016 mmol), $Pd_2(dba)_3$ (1.18 mg, 0.18×10^{-3} mmol), P(o-tolyl)$_3$ (1.00 mg, 5.06×10^{-3} mmol), and toluene (2 mL) were added. The tube was purged with nitrogen and sealed under nitrogen flow and stirred at 110 °C for 3 days. Then the reaction mixture was cooled down to room temperature and precipitated into 200 mL of methanol. The solid residue was re-dissolved in chloroform (2 mL) and

added slowly to methanol (20 mL). The precipitates were collected by filtration, washed with methanol, and dried in vacuum, leading to a deep blue solid as the product DYF-V (50 mg, yield 86%). ^{1}H NMR (CDCl$_3$, 400 MHz) δ 9.15 (d, *J* = 25.2 Hz, 4H), 8.59 (m, 4H), 8.06 (s, 2H), 7.67 (m, 4H), 4.83 (s, 8H), 3.18 (d, *J* = 49.2 Hz, 8H), 2.16 (s, 4H), 1.88 (s, 8H), 1.03 (m, 268H). ^{13}C NMR (101 MHz, CDCl3) *δ* 184.59, 181.13, 153.85, 146.99, 146.79, 146.07, 144.36, 136.96, 136.89, 136.80, 136.68, 134.61, 134.38, 132.61, 130.87, 128.81, 126.67, 119.37, 119.24, 118.64, 114.56, 114.19, 113.00, 112.12, 67.43, 31.30, 31.29, 31.17, 29.96, 29.76, 29.26, 29.11, 29.07, 29.02, 28.98, 28.94, 28.91, 28.82, 28.73, 28.71, 28.58, 24.80, 22.07. MS (MALDI-TOF) [M] calcd. for ($C_{214}H_{270}F_6N_{16}O_4S_{10}$):3565.21. Found: 3564.22.

2,2'-((2Z,2'Z)-(((((2Z,2'Z)-((E)-ethene-1,2-diyl)bis(1-(dicyanomethylene)-4,6-difluoro-3-oxo-1,3-dihydro-2H-indene-5-yl-2-ylidene))bis(methanylylidene))bis(12,13-bis(2-octyldodecyl)-3,9-diundecyl-12,13-dihydro-[1,2,5]thiadiazolo[3,4-e]thieno[2'',3'':4',5']thieno[2',3':4,5]pyrrolo[3,2-g]thieno[2',3':4,5]thieno[3,2-b]indole-10,2-diyl))bis(methanylylidene))bis(5,6-difluoro-3-oxo-2,3-dihydro-1H-indene-2,1-diylidene))dimalononitrile (DY2F-V) (50 mg, yield 86%) was prepared according to the same method as DYF-V. ^{1}H NMR (CDCl$_3$, 400 MHz) δ 9.16 (d, *J* = 6.0 Hz, 4H), 8.57 (m, 2H), 8.39 (d, J = 10.4 Hz, 2H), 7.77 (s, *J* = 7.6 Hz, 2H), 7.67 (m, 2H), 4.79 (d, *J* = 6.4 Hz, 8H), 3.23 (m, 8H), 2.15 (s, 4H), 1.88 (d, *J* = 7.2 Hz, 8H), 1.54 (s, 8H), 1.03 (m, 232H). ^{13}C NMR (101 MHz, CDCl$_3$) *δ* 153.76, 153.29, 146.88, 144.73, 144.64, 137.33, 137.16, 135.39, 133.48, 132.93, 132.72, 113.07, 31.30, 31.24, 31.17, 29.27, 29.15, 29.05, 29.02, 29.00, 28.95, 28.93, 28.93, 28.82, 28.77, 28.73, 28.59, 22.07, 22.00, 13.49. MS (MALDI-TOF) [M] calcd. for ($C_{214}H_{268}F_8N_{16}O_4S_{10}$):3601.19. Found: 3600.10.

Supplementary Figures.

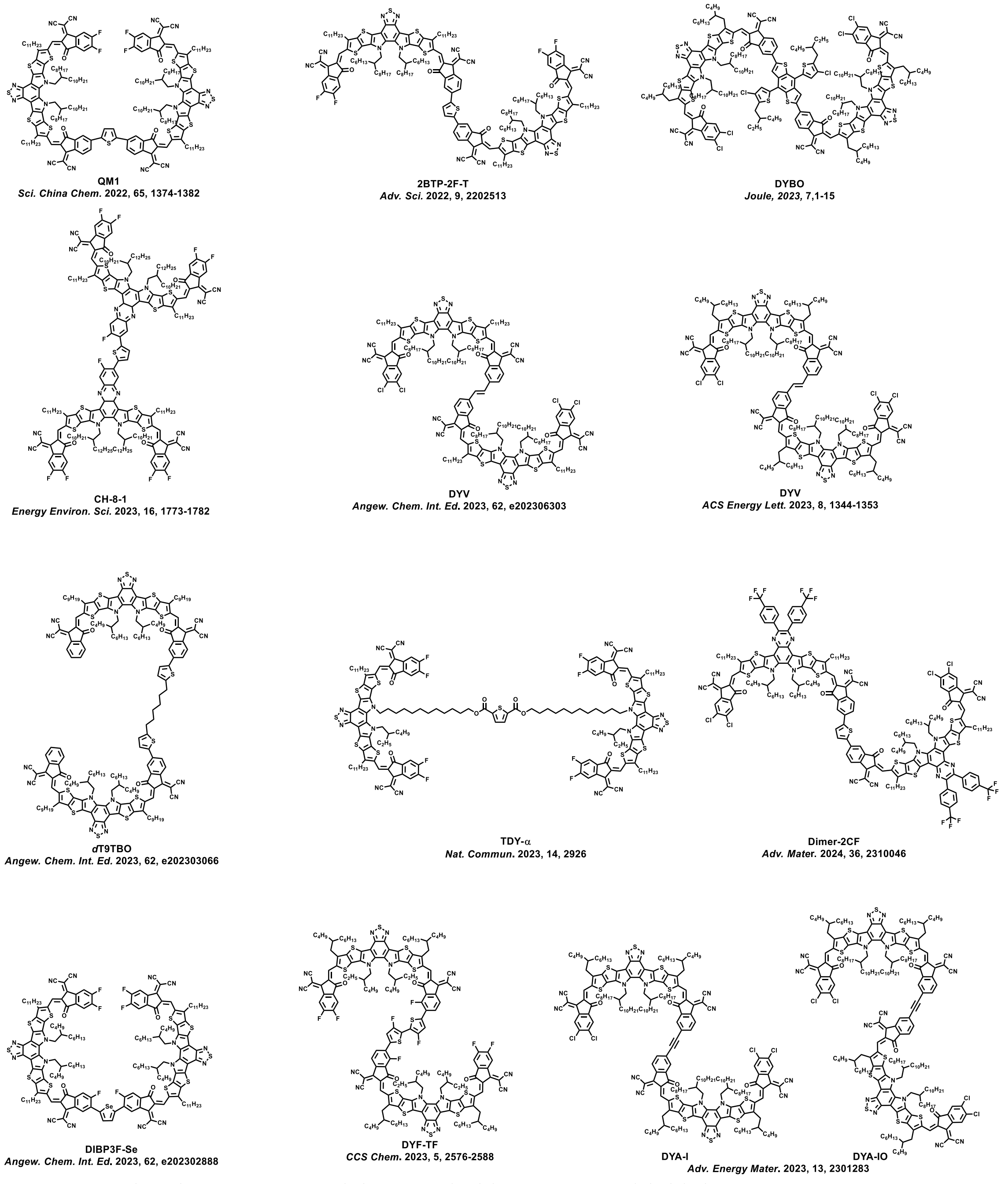

Figure S1. Molecular structures of the DAs in binary OPV with high PCEs over 17%.

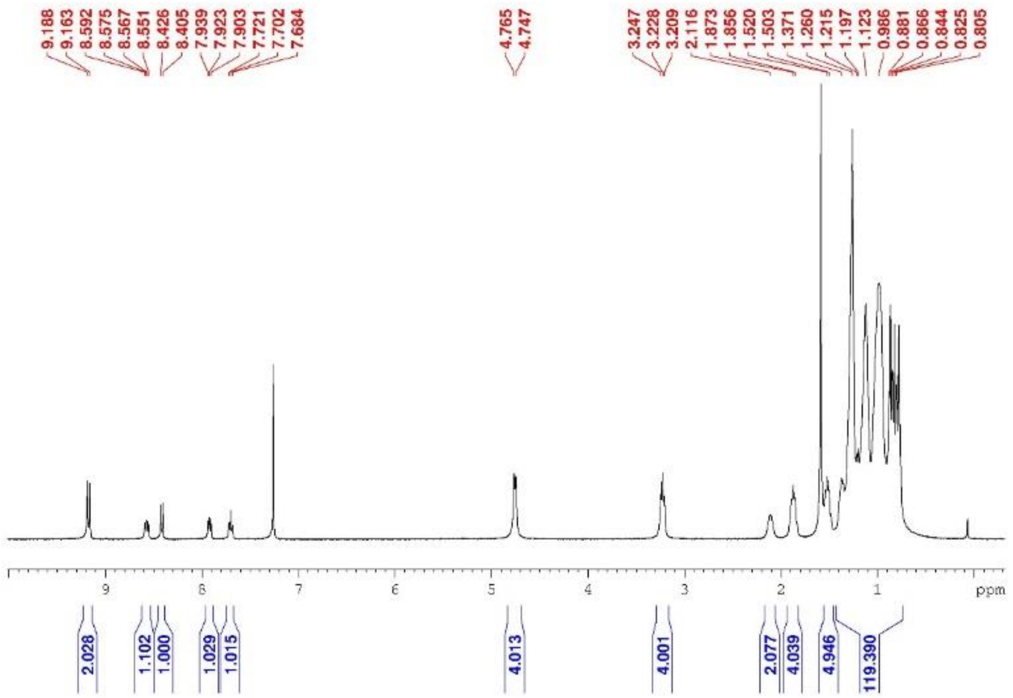

Figure S2. ^{1}H NMR spectrum of compound **5b**.

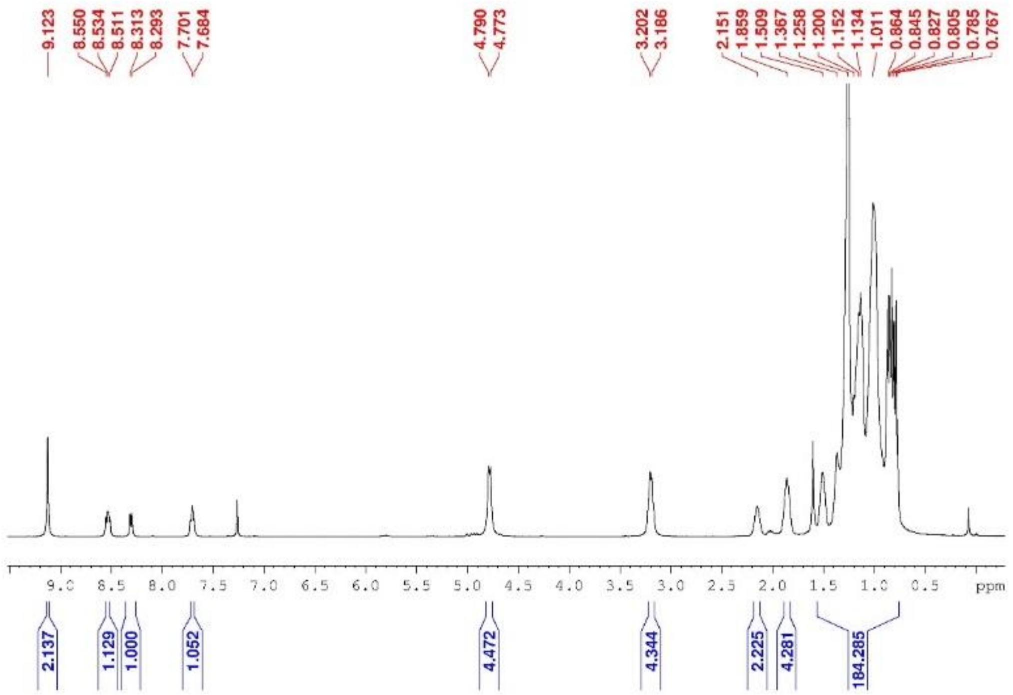

Figure S3. ^{1}H NMR spectrum of compound **5c**.

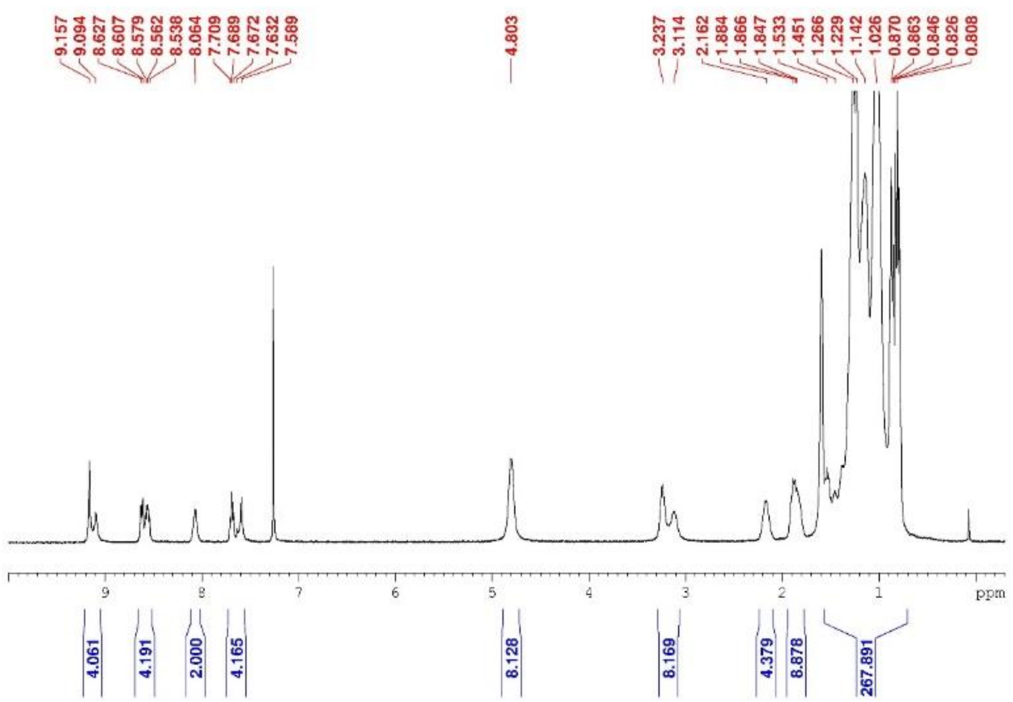

Figure S4. ^{1}H NMR spectrum of compound DYF-V.

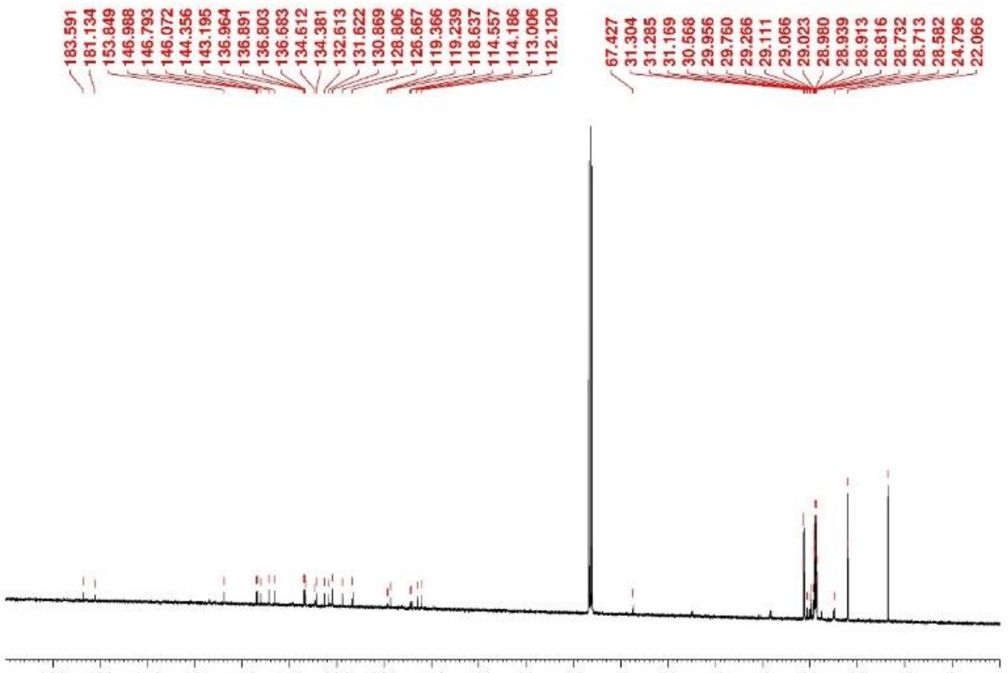

Figure S5. ^{13}C NMR spectrum of compound DYF-V.

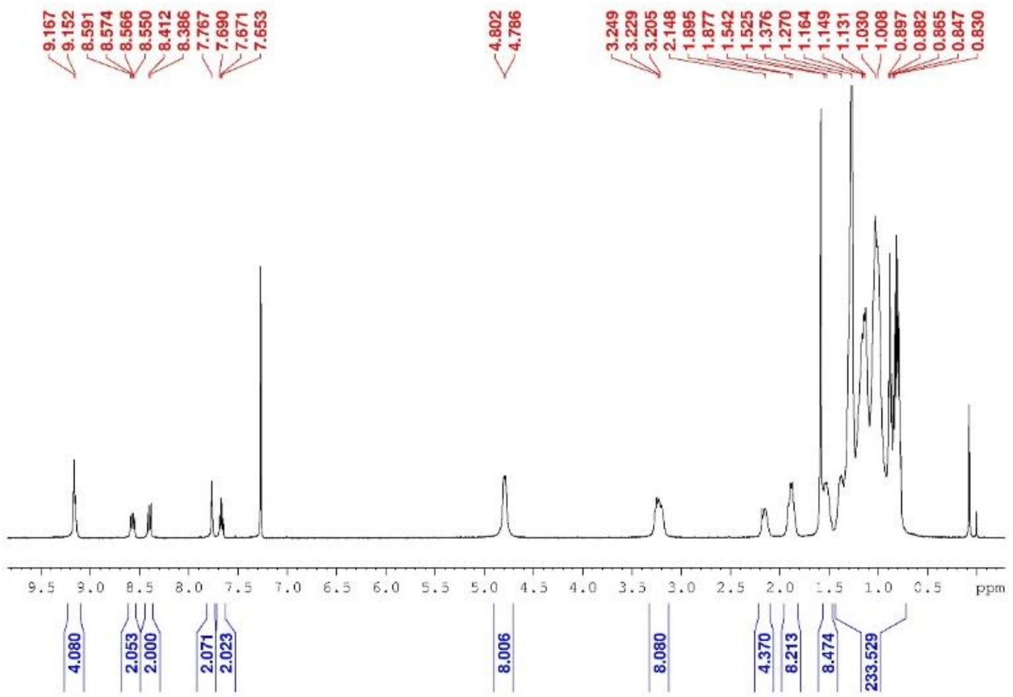

Figure S6. ^{1}H NMR spectrum of compound DY2F-V.

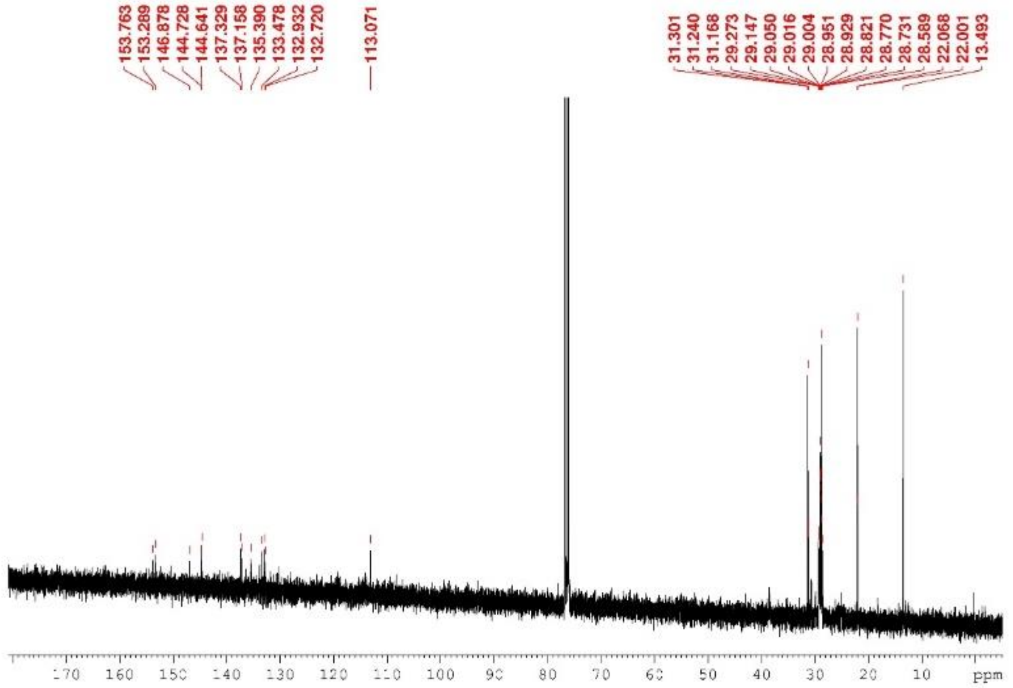

Figure S7. ^{13}C NMR spectrum of compound DY2F-V.

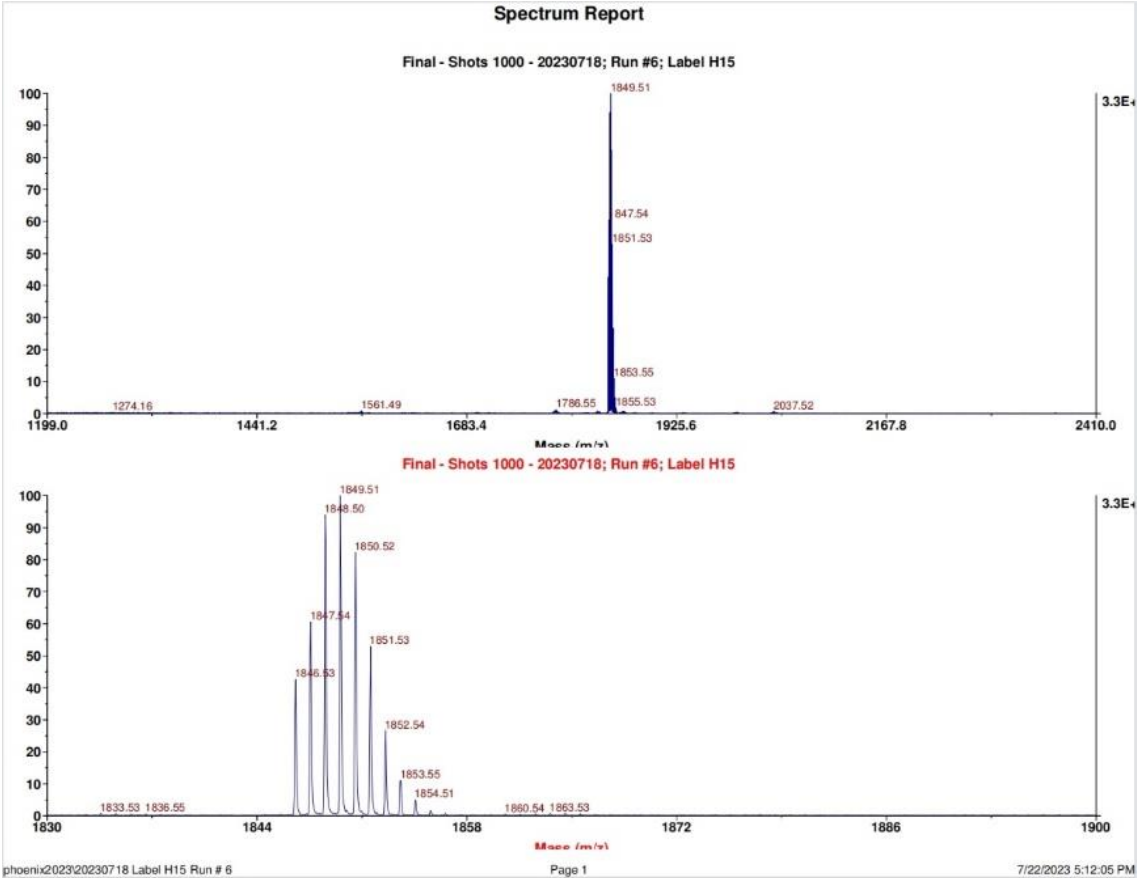

Figure S8. High resolution mass spectra of **5b**.

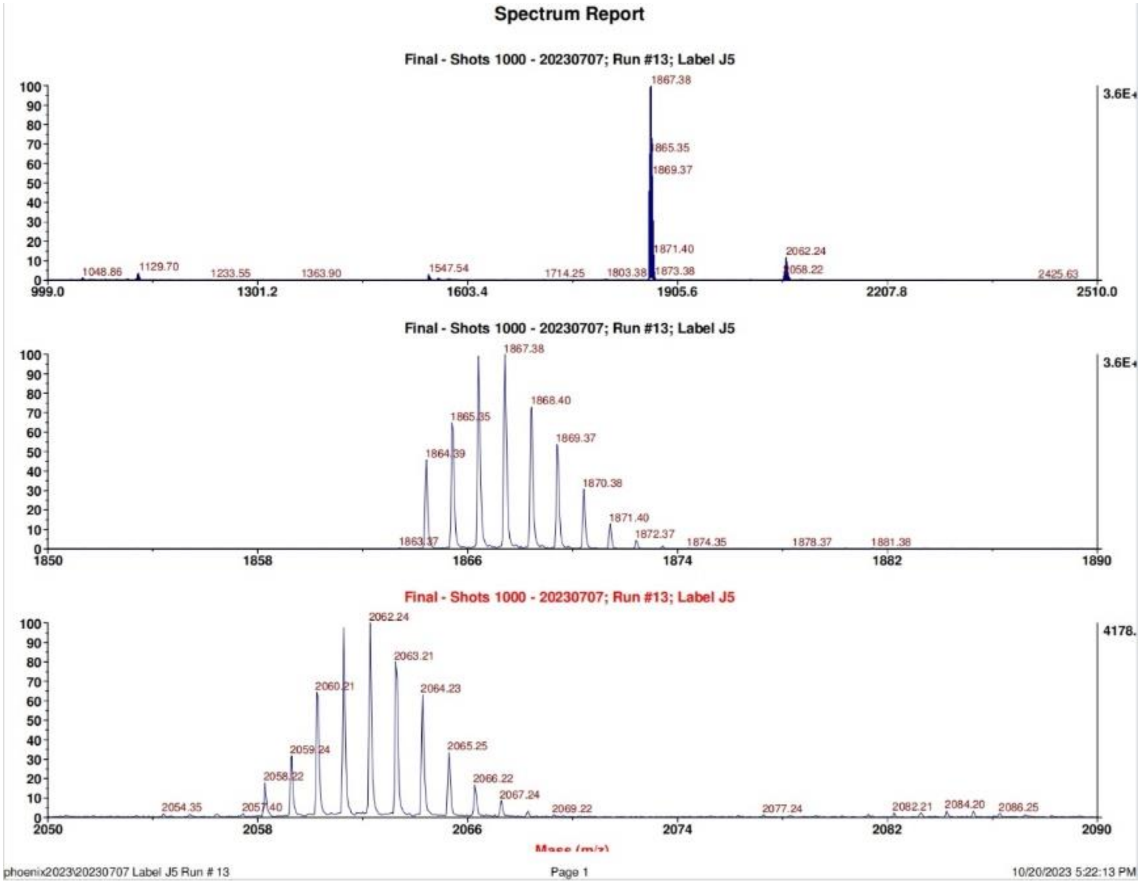

Figure S9. High resolution mass spectra of **5c**.

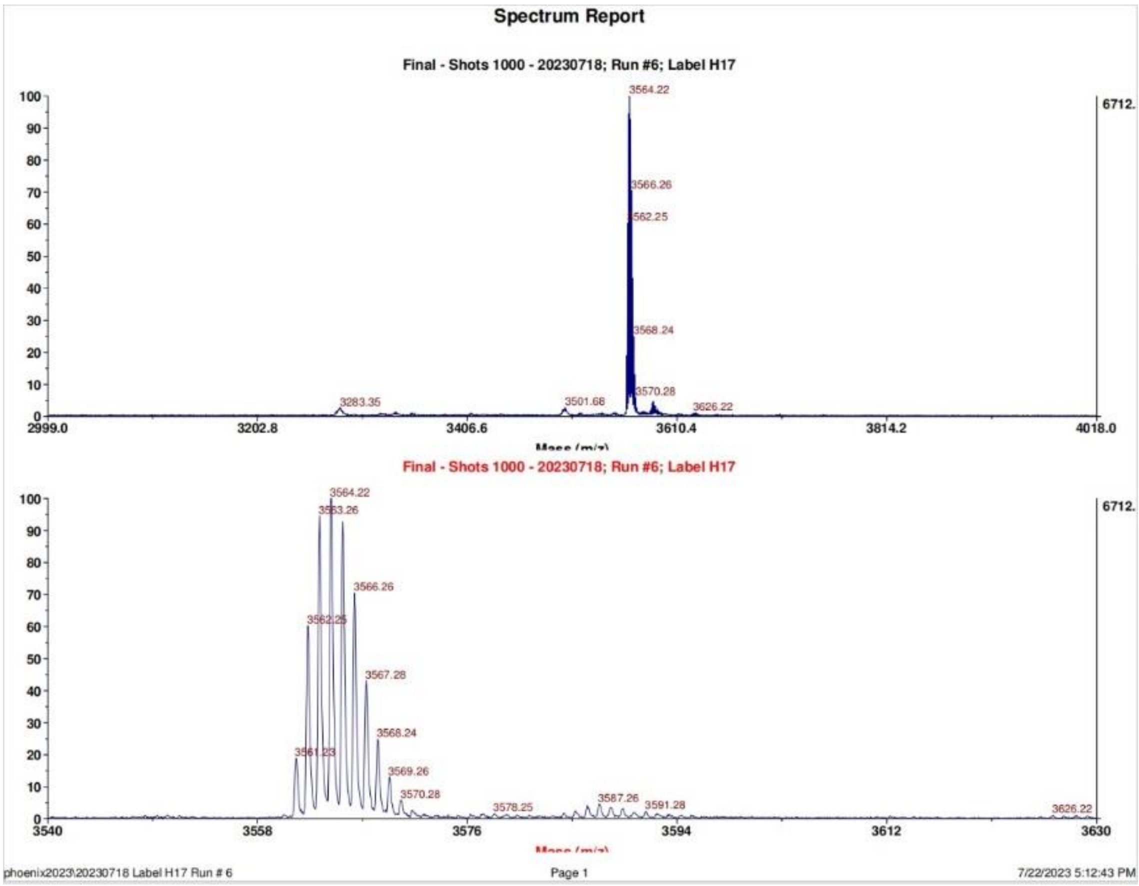

Figure S10. High resolution mass spectra of DYF-V.

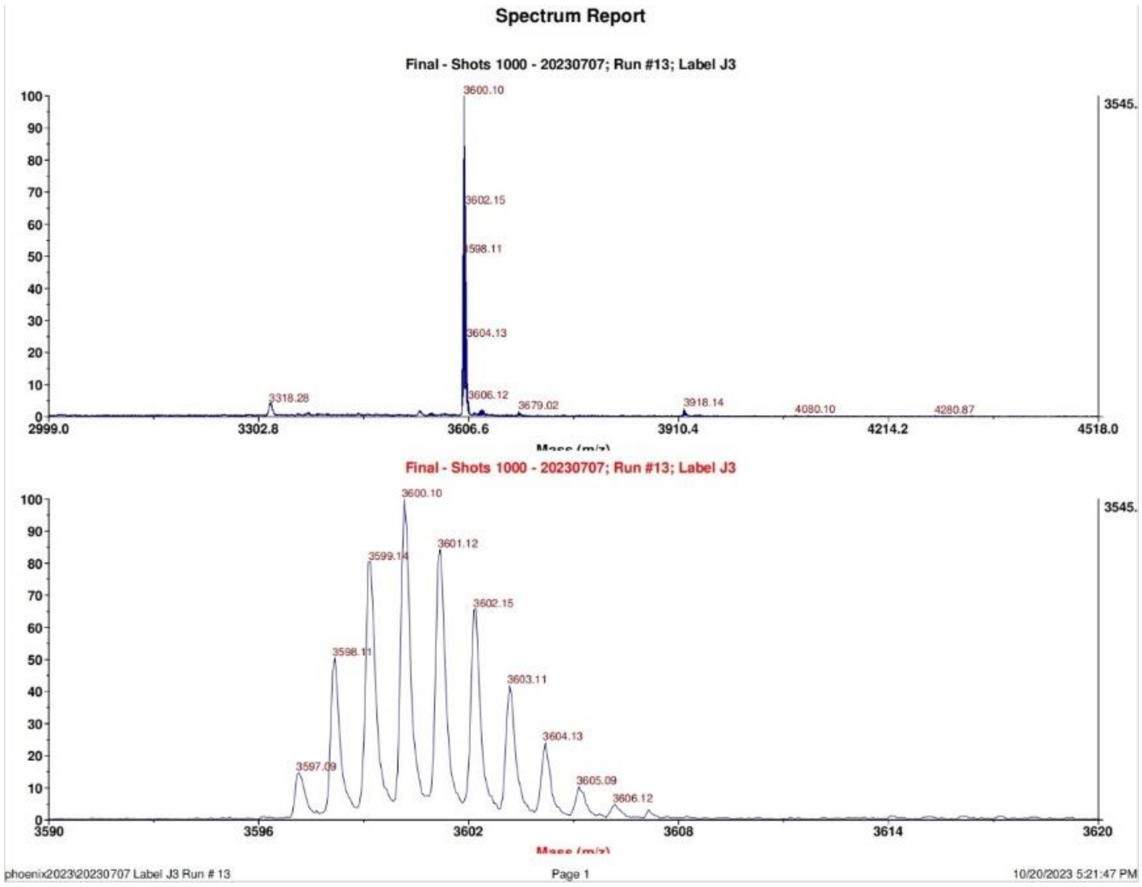

Figure S11. High resolution mass spectra of DY2F-V.

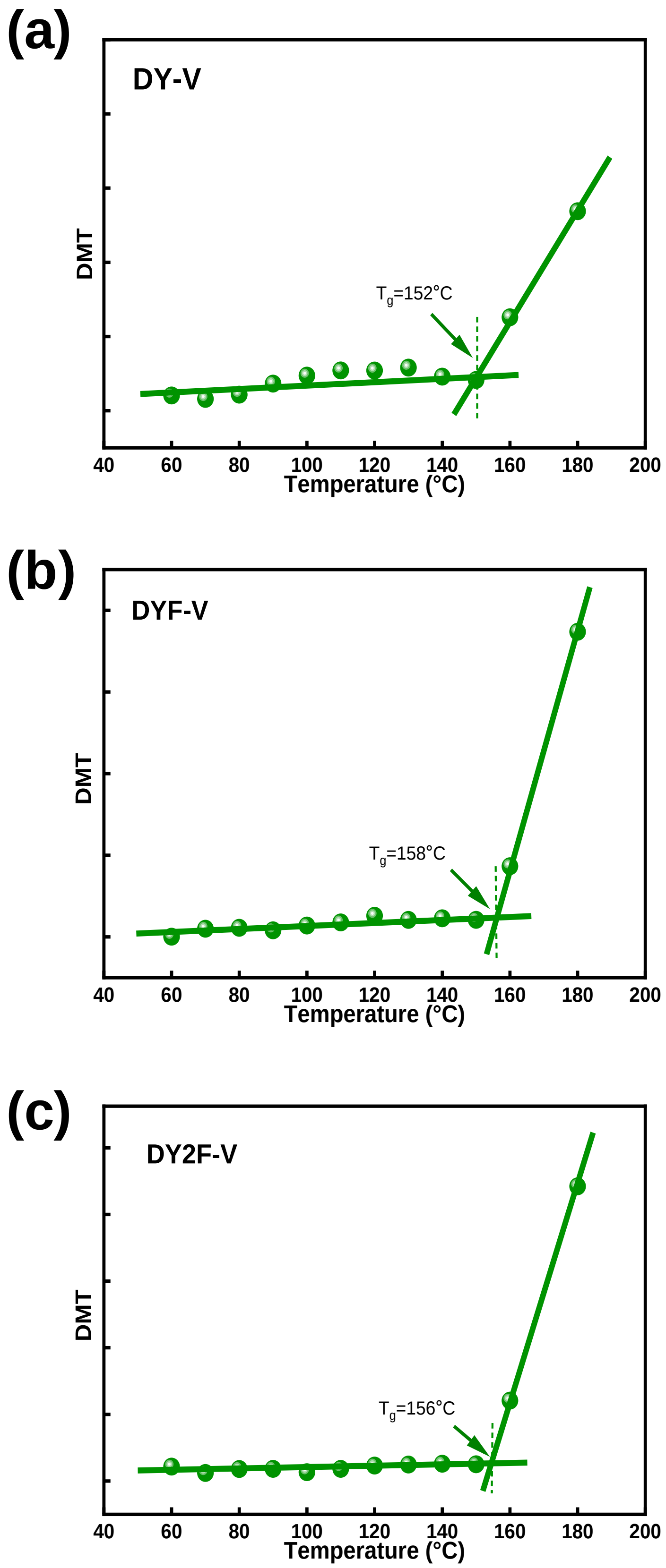

Figure S12. Plots of the DMT of DY-V, DYF-V, and DY2F-V films as a function of annealing temperature.

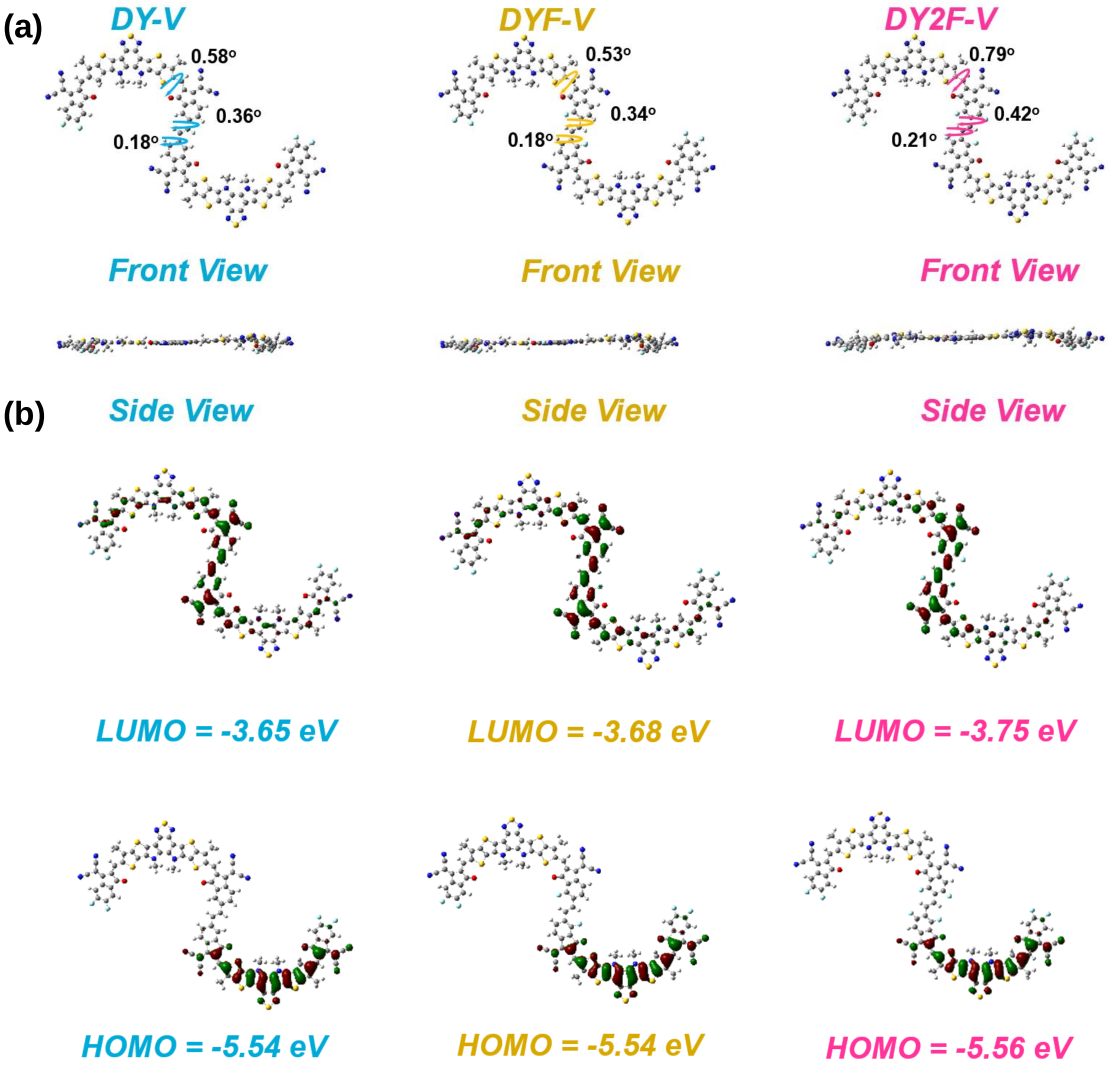

Figure S13. (a)The optimized molecular geometry of DY-V, DYF-V and DY2F-V calculated by a DFT method at the B3LYP/6-31G(d,p) set and (b) the calculated frontier orbitals of the DY-V, DYF-V and DY2F-V dimers through the DFT method.

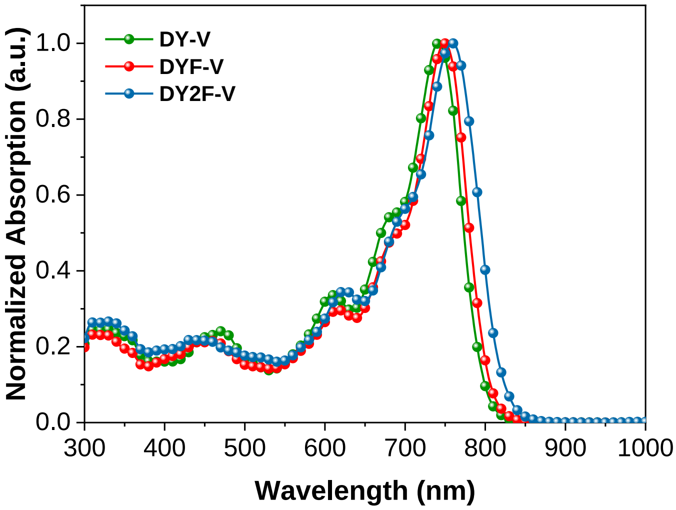

Figure S14. Solution absorption spectra of DY-V, DYF-V and DY2F-V.

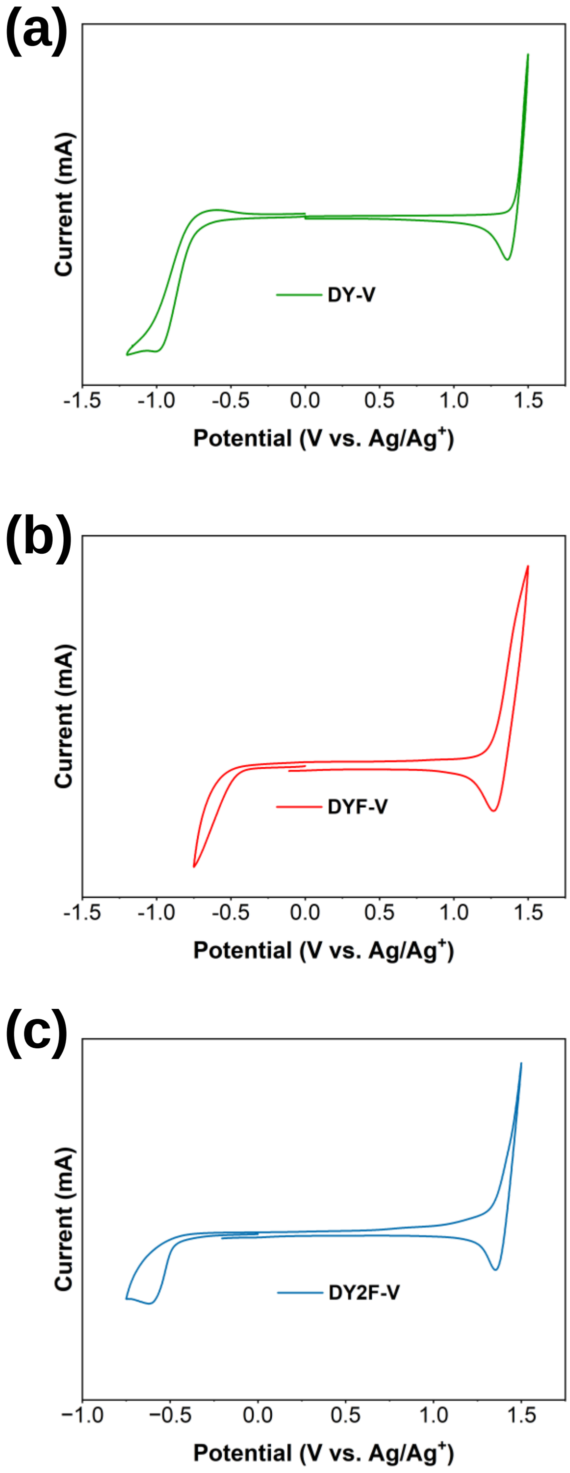

Figure S15. Cyclic voltammogram of DY-V, DYF-V and DY2F-V film in acetonitrile solution with 0.1 mol L^{-1} n-Bu_4NPF_6 at a scan rate of 100 mV s^{-1}. The HOMO/LUMO energy levels were calculated from the onset oxidation potential and the onset reduction potential vs FC/FC+, using the equation $E_{HOMO} = -(4.80 + E_{onset\ vs\ FC/FC+}{}^{ox})$, $E_{LUMO} = -(4.80 + E_{onset\ vs\ FC/FC+}{}^{re})$.

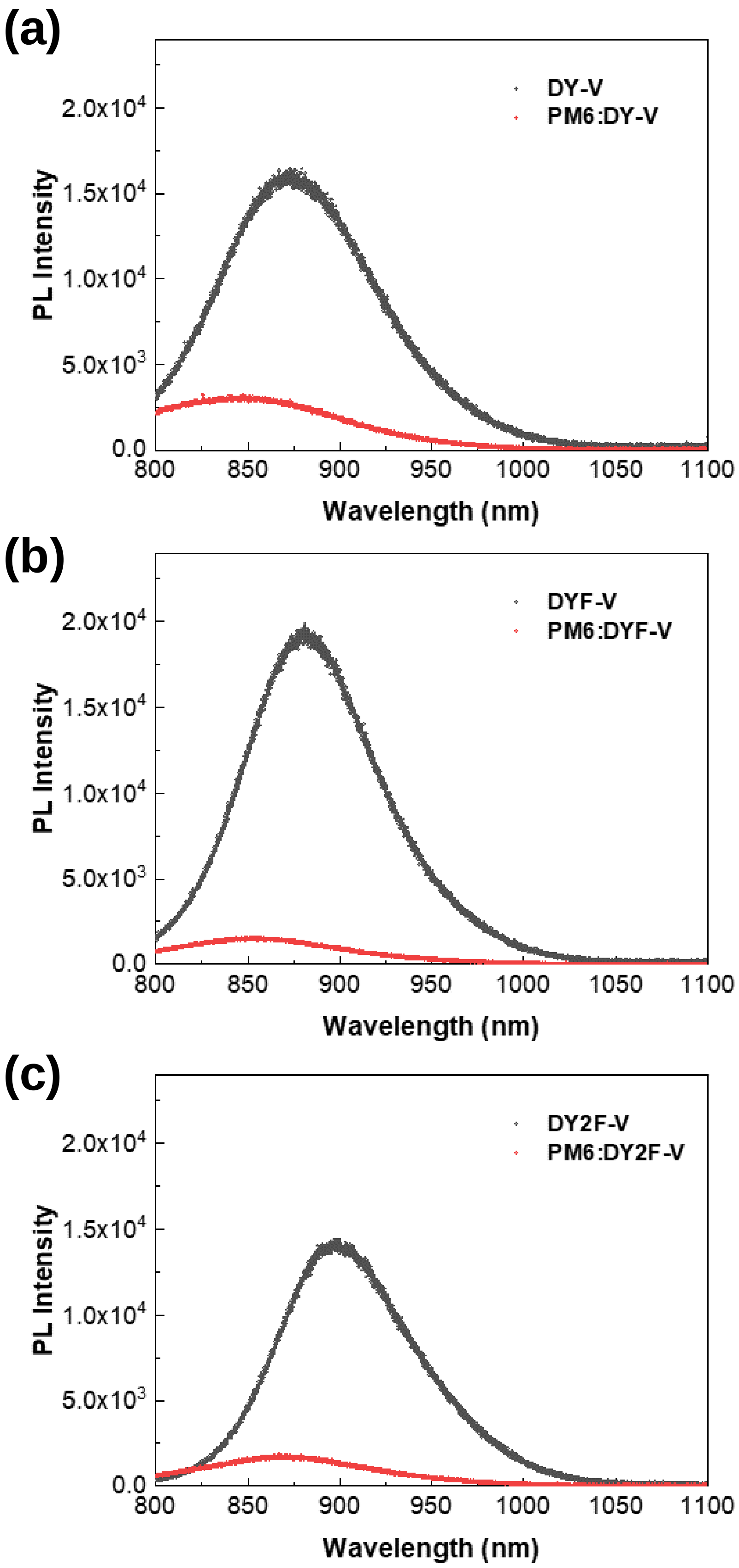

Figure S16. Photoluminescence (PL) spectra of DAs films and their blend films of PM6:DY-V, PM6:DYF-V and PM6:DY2F-V.

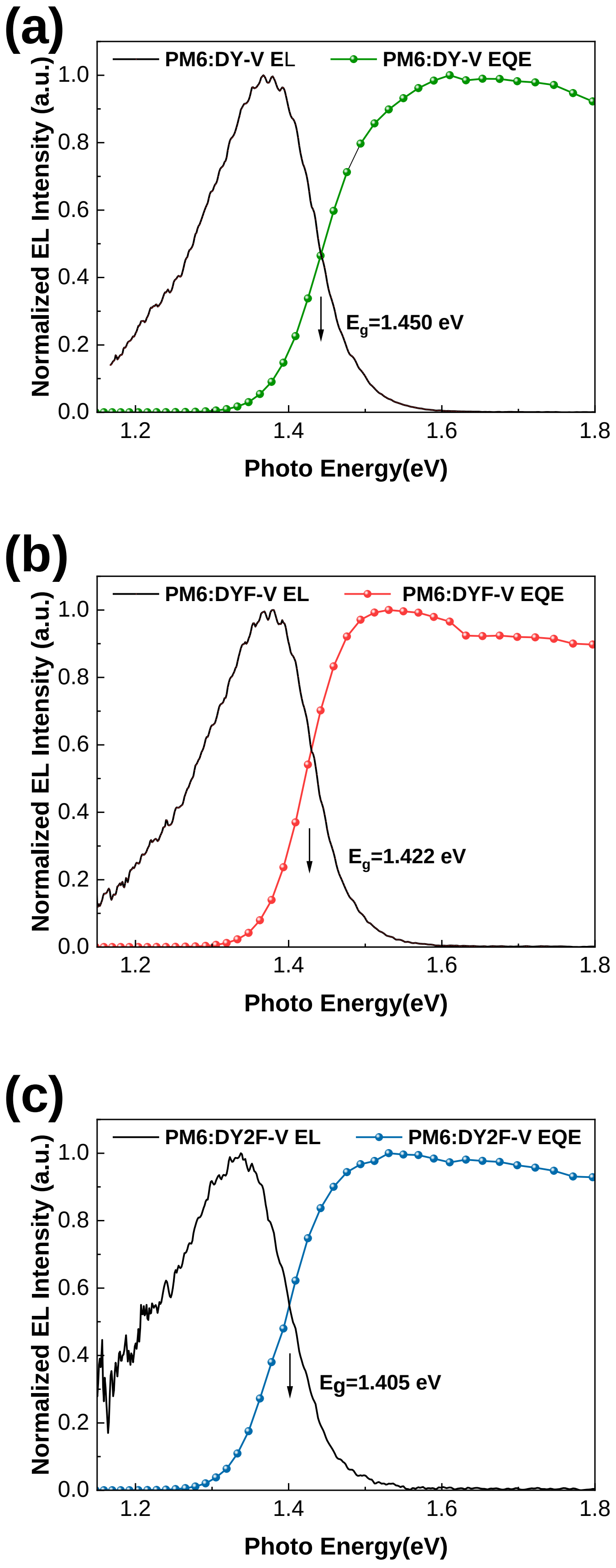

Figure S17. Normalized electroluminescence (EL) and external quantum efficiency (EQE) spectra of PM6:DY-V, PM6:DYF-V and PM6:DY2F-V for accurate bandgap calculation.

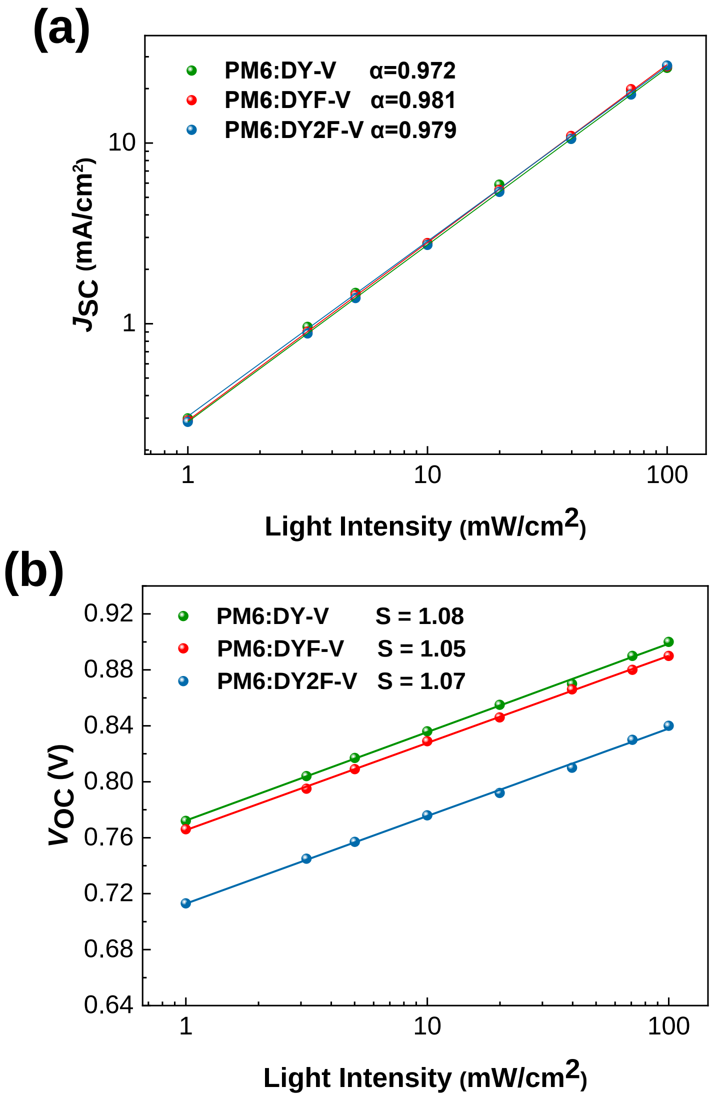

Figure S18. Dependence of (a) J_{SC} and (b) V_{OC} on different light intensity (P_{light}) of the corresponding PM6:DY-V, PM6:DYF-V, PM6:DY2F-V-based devices.

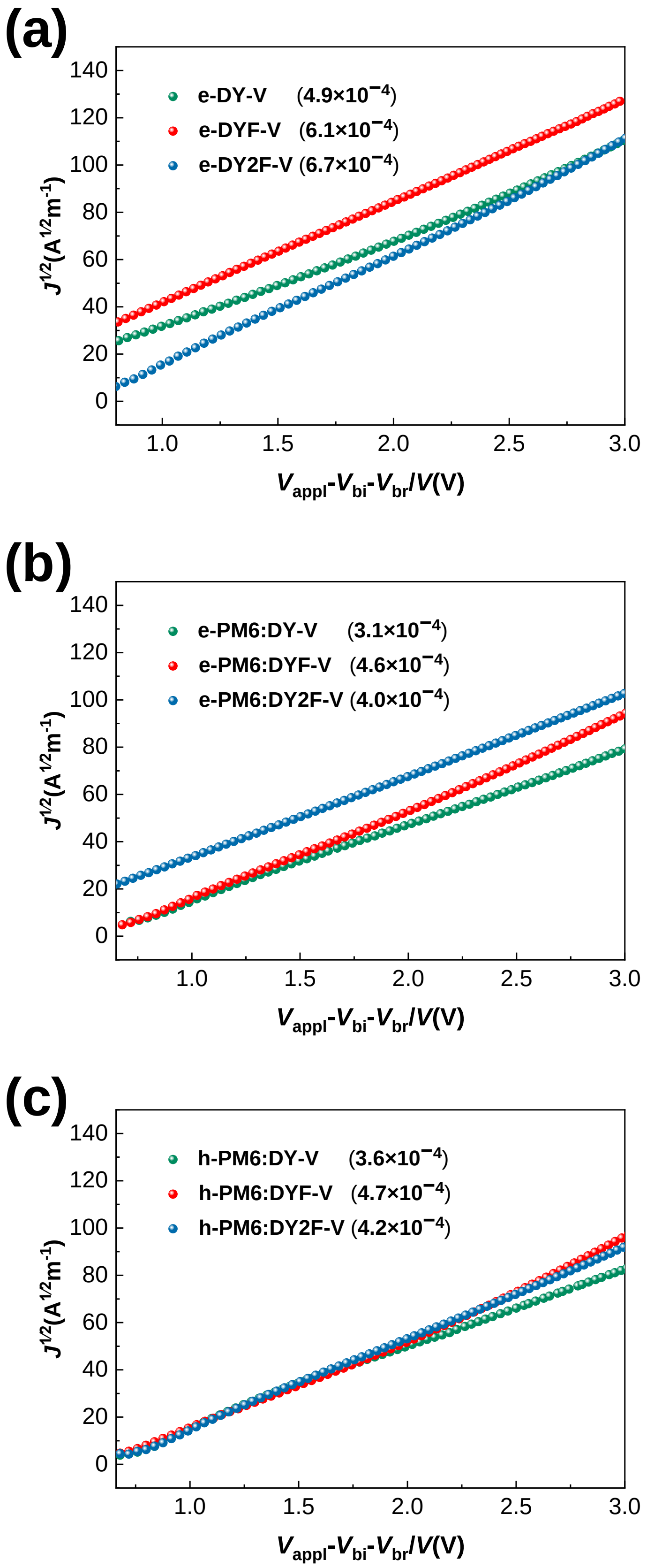

Figure S19. $J^{1/2}$~V characteristics of (a) electron-only devices of the DY-V, DYF-V and DY2F-V films. (b) hole-only devices and (c) electron-only devices of the PM6:DY-V, PM6:DYF-V and PM6:DY2F-V blend films.

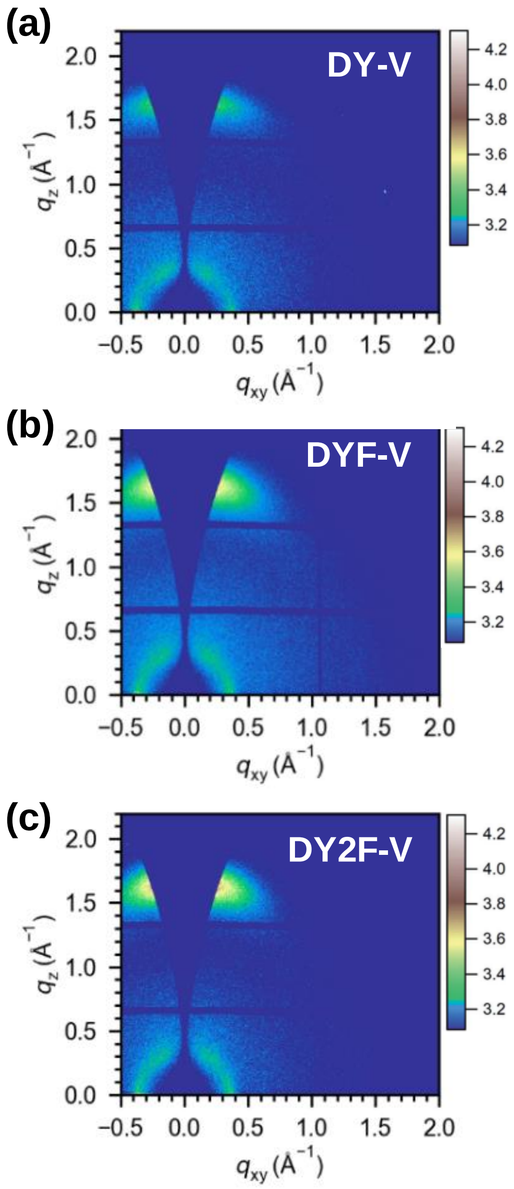

Figure S20. 2D GIWAXS patterns of DY-V, DYF-V and DY2F-V pristine films.

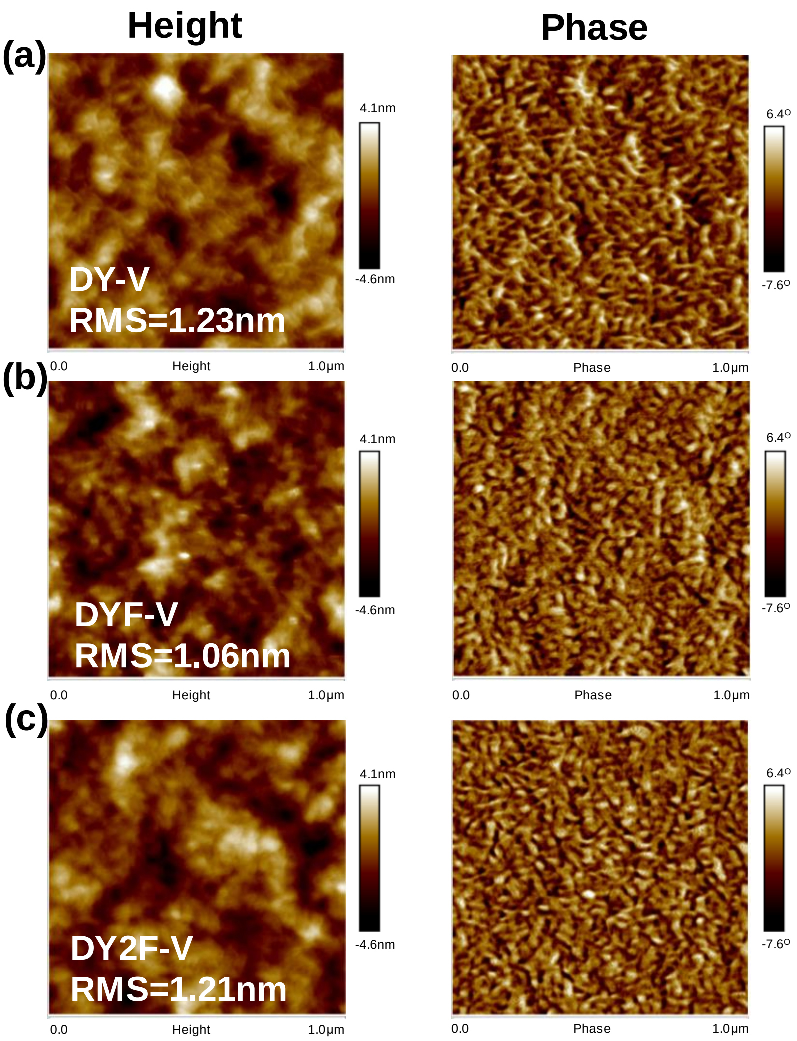

Figure S21. Height (left) and phase images (right) of PM6:DY-V, PM6:DYF-V and PM6:DY2F-V. blend films obtained by AFM tapping mode.

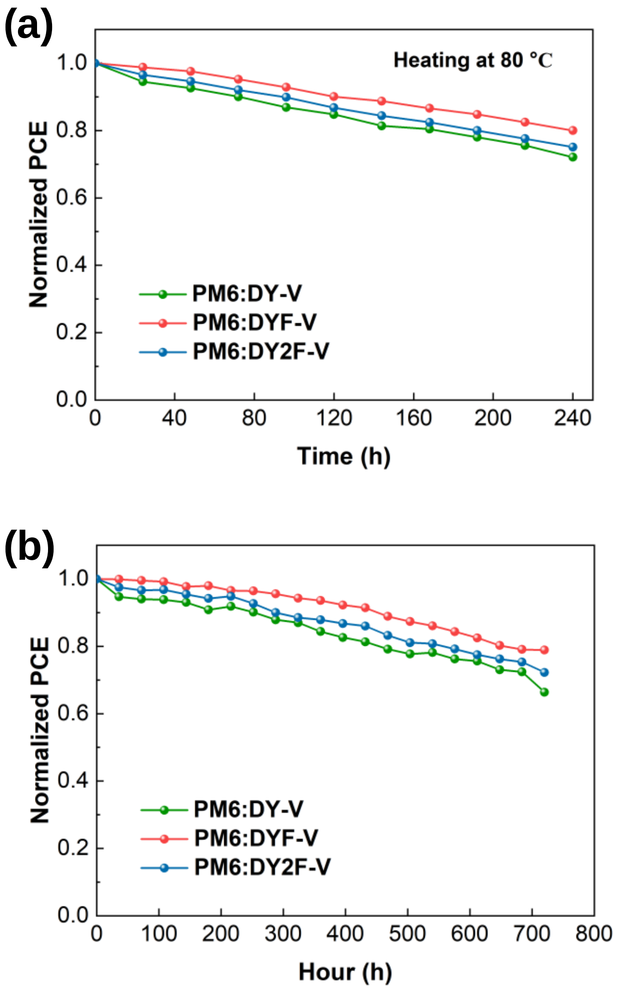

Figure S22. Variation of normalized PCE of the relevant devices. (a) Thermal stability plots and (b) Light-soaking stability plots of encapsulated devices based on PM6:DY-V, PM6:DYF-V and PM6:DY2F-V.

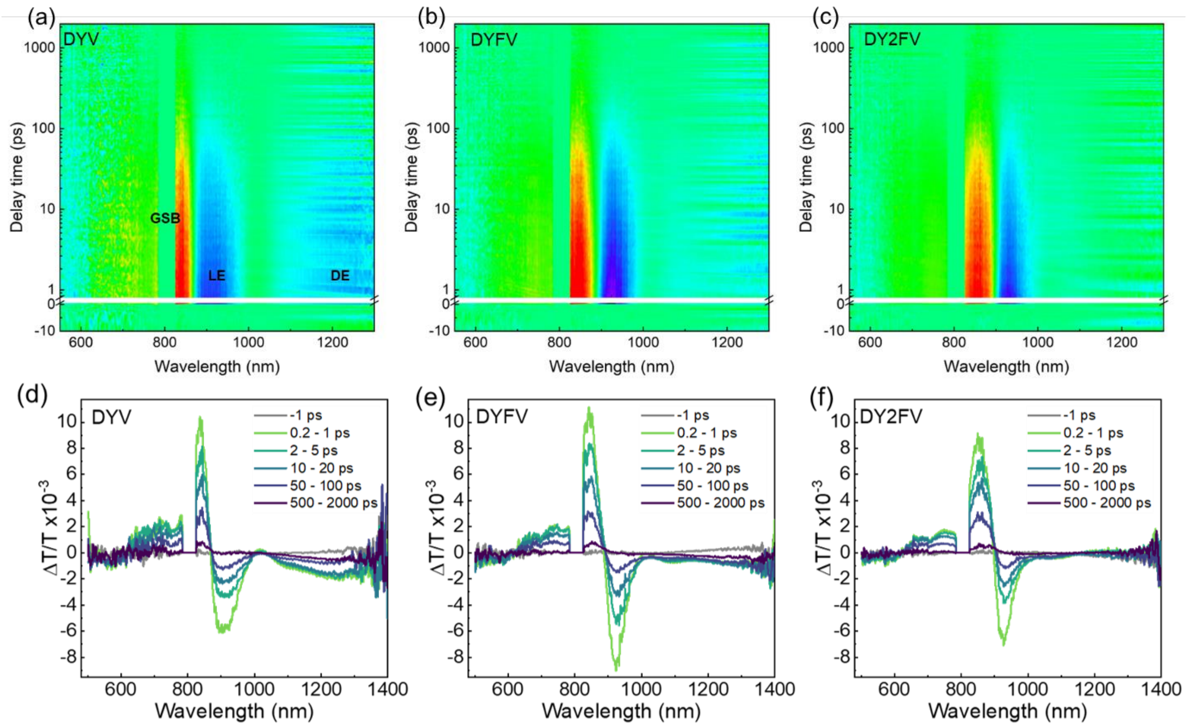

Figure S23. TA contour plots of neat dimer-V films under 800 nm excitation: (a) DYV, (b) DYFV, and (c) DY2FV; GBS, LE, and DE denote ground-state bleaching, local exciton, and delocalized state, correspondingly. Transient absorption spectra of neat acceptor (d-e) films at different delay times after 800-nm photoexcitaion.

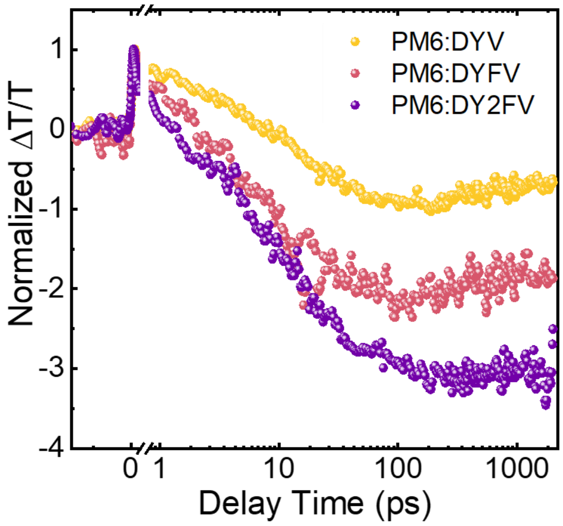

Figure S24. TA kinetics of electro absorption band.

Supplementary Tables.

Table S1. Summary of photovoltaic performance parameters of the DAs binary OPV with high PCEs over 17%.

	V_{OC} [V]	J_{SC} [mA/cm^2]	FF [%]	PCE [%]	Ref.
**PM6:DYF-V**	**0.89**	**26.62**	**78.56**	**18.63**	**This work**
**PM6:DY2F-V**	**0.84**	**26.83**	**76.17**	**17.25**	**This work**
**PM6:QM1**	0.91	25.23	74.01	17.05	[4]
**PM6:*d*BTICγ-EH**	0.92	21.43	73.28	14.48	[5]
**PM6:2BTP-2F-T**	0.91	25.50	72.28	18.19	[6]
**PM6:CH-8-1**	0.92	24.89	74.20	17.05	[7]
**PM6:DYV**	0.91	25.97	76.22	18.01	[8]
**D18:DYF-TF**	0.94	25.82	75.30	18.26	[9]
**PM6:DIBP3F-Se**	0.92	25.92	76.10	18.09	[10]
**PM6:DYBO**	0.97	24.62	75.80	18.08	[11]
**PM6:DYV**	0.93	25.64	78.00	18.60	[12]
**D18/BTP-eC9:*d*T9TBO**	0.88	27.79	75.90	18.57	[13]
**PM6:TDY-α**	0.86	26.90	78.00	18.10	[14]
**PM6:Dimer-2CF**	0.89	25.27	80.62	18.12	[15]
**PM6:Dimer-2CF (HTL:2PACz)**	0.90	26.39	80.03	19.02	[15]
**D18:DYA-I**	0.94	25.67	78.00	18.83	[16]
**D18:DYA-IO**	0.95	24.10	76.00	17.54	[16]

Table S2. The parameters of exciton dissociation efficiency and charge collection efficiency.

Active Layer	J_{sat}(mA/cm^2)	J_{ph}[a](mA/cm^2)	J_{ph}[b](mA/cm^2)	η_{diss}(%)	η_{coll}(%)
**PM6:DY-V**	26.61	26.04	23.13	97.9	86.9
**PM6:DYF-V**	26.99	26.62	24.62	98.6	91.2
**PM6:DY2F-V**	27.27	26.83	24.48	98.4	89.8

[a] Under the maximal power output condition.
[b] Under short circuit condition.

Table S3. Detailed energy loss of PM6: DY-V, PM6:DYF-V and PM6: DY2F-V-based DAs.

Blend	E_g (eV)	qV_{OC}^{SQ} (eV)[a]	qV_{OC}^{rad} (eV)[b]	qV_{loss}	ΔE_1 (eV)	ΔE_2 (eV)[c]	ΔE_3 (eV)[d]
**PM6:DY-V**	1.450	1.185	1.119	0.553	0.265	0.066	0.223
**PM6:DYF-V**	1.422	1.160	1.102	0.533	0.262	0.058	0.213
**PM6:DY2F-V**	1.404	1.128	1.063	0.560	0.276	0.065	0.219

[a] V_{OC}^{SQ}: Schokley-Queisser limit to V_{OC}.
[b] V_{OC}^{rad}: radiative limit to V_{OC}, measured using EQE_{EL}.
[c] ΔE_2 ((qV_{OC}^{SQ} -qV_{OC}^{rad}): voltage losses due to non-ideal absorption (it was calculated from EL and FTPS measurements).
[d] ΔE_3 ($q\Delta V_{OC}^{non\text{-}rad}$): voltage losses due to non-radiative recombination only.

Table S4. The parameters of hole mobilities and electron mobilities of pristine DY-V, DYF-V, DY2F-V and PM6: DY-V, PM6: DYF-V, PM6: DY2F-V blends.

Material Combination	Electron mobility, μ_e (10^{-4} cm^{-2} V^{-1} s^{-1})	Hole mobility, μ_h (10^{-4} cm^{-2} V^{-1} s^{-1})	μ_h/μ_e
**DY-V**	4.9×10^{-4}		
**DYF-V**	6.1×10^{-4}		
**DY2F-V**	6.7×10^{-4}		
**PM6:DY-V**	3.1×10^{-4}	3.6×10^{-4}	1.16
**PM6:DYF-V**	4.6×10^{-4}	4.7×10^{-4}	1.02
**PM6:DY2F-V**	4.0×10^{-4}	4.2×10^{-4}	1.05

Table S5. Summary of pristine films and blend films morphology parameters extracted from the GIWAXS measurements.

**Material**	**Peak location (010) (Å^{-1})**	**π-π stack spacing (Å)**	**CCL (010) (Å)**
**DY-V**	1.69	3.72	21.57
**DYF-V**	1.64	3.71	21.74
**DY2F-V**	1.64	3.71	26.57
**PM6:DY-V**	1.69	3.71	25.69
**PM6:DYF-V**	1.67	3.69	28.69
**PM6:DY2F-V**	1.66	3.68	29.75

Table S6. The fitting results for GSBA, GSBD, and PH kinetics of the acceptor (GSB_A), ground-state bleaching of the donor (GSB_D), and hole polaron (P_H) kinetics.

**Material**	**PM6:DY-V**	**PM6:DYF-V**	**PM6:DY2F-V**
		GSB_A	
**τ_1 (ps)**	0.76	0.36	0.85
**τ_2 (ps)**	12.59	5.28	13.45
**τ_3 (ps)[a]**	9814	7706	13627
		GSB_D	
**τ_1 (ps)**	0.41	0.60	0.40
**τ_2 (ps)**	10.60	10.45	12.06
		P_H	
**τ_1 (ps)**	1.89	1.40	0.90
**τ_2 (ps)**	17.27	12.50	14.77

[a]These decay times corresponds to depopulation of LE state due to radiative recombination. Since the total decay time exceeds the delay time range of the experimental setup, the values might be overestimated.

References

[1] R. Wang, C. Zhang, Q. Li, Z. Zhang, X. Wang, M. Xiao, *J. Am. Chem. Soc.,* 2020, **142**, 12751-12759.

[2] G. Zhang, X.-K. Chen, J. Xiao, P. C. Y. Chow, M. Ren, G. Kupgan, X. Jiao, C. C. S. Chan, X. Du, R. Xia, Z. Chen, J. Yuan, Y. Zhang, S. Zhang, Y. Liu, Y. Zou, H. Yan, K. S. Wong, V. Coropceanu, N. Li, C. J. Brabec, J.-L. Bredas, H.-L. Yip, Y. Cao, *Nat. Commun.,* 2020, **11**, 3943.

[3] X. Zou, H. Yu, Z. Qi, B. Liu, Z. Xing, C. C. S. Chan, P. C. Y. Chow, D. Pan, H. Yan, K. S. Wong, *Sol. RRL,* 2022, **6**, 2200169.

[4] W. Liu, J. Yuan, C. Zhu, Q. Wei, S. Liang, H. Zhang, G. Zheng, Y. Hu, L. Meng, F. Gao, Y. Li, Y. Zou, *Sci. China Chem.,* 2022, **65**, 1374-1382.

[5] H. Wang, C. Cao, H. Chen, H. Lai, C. Ke, Y. Zhu, H. Li, F. He, *Angew. Chem. Int. Ed.,* 2022, **61**, e202201844.

[6] L. Zhang, Z. Zhang, D. Deng, H. Zhou, J. Zhang, Z. Wei, *Adv. Sci.,* 2022, **9**, 2202513.

[7] H. Chen, Z. Zhang, P. Wang, Y. Zhang, K. Ma, Y. Lin, T. Duan, T. He, Z. Ma, G. Long, C. Li, B. Kan, Z. Yao, X. Wan, Y. Chen, *Energy Environ. Sci.,* 2023, **16**, 1773-1782.

[8] H. Fu, M. Zhang, Y. Zhang, Q. Wang, Z. a. Xu, Q. Zhou, Z. Li, Y. Bai, Y. Li, Z. G. Zhang, *Angew. Chem. Int. Ed.,* 2023, **62**, e202306303.

[9] X. Gu, Y. Wei, N. Yu, J. Qiao, Z. Han, Q. Lin, X. Han, J. Gao, C. Li, J. Zhang, X. Hao, Z. Wei, Z. Tang, Y. Cai, X. Zhang, H. Huang, *CCS Chem.,* 2023, **5**, 2576-2588.

[10] J. Wu, Z. Ling, L. R. Franco, S. Y. Jeong, Z. Genene, J. Mena, S. Chen, C. Chen, C. M. Araujo, C. F. N. Marchiori, J. Kimpel, X. Chang, F. H. Isikgor, Q. Chen, H. Faber, Y. Han, F. Laquai, M. Zhang, H. Y. Woo, D. Yu, T. D. Anthopoulos, E. Wang, *Angew. Chem. Int. Ed.,* 2023, **62**, e202302888.

[11] C. Sun, J.-W. Lee, C. Lee, D. Lee, S. Cho, S.-K. Kwon, B. J. Kim, Y.-H. Kim, *Joule,* 2023, **7**, 416-430.

[12] J.-W. Lee, C. Sun, C. Lee, Z. Tan, T. N.-L. Phan, H. Jeon, D. Jeong, S.-K. Kwon, Y.-H. Kim, B. J. Kim, *ACS Energy Lett.,* 2023, **8**, 1344-1353.

[13] F. Qi, Y. Li, R. Zhang, F. R. Lin, K. Liu, Q. Fan, A. K. Y. Jen, *Angew. Chem. Int. Ed.,* 2023, **62**, e202303066

[14] Y. Bai, Z. Zhang, Q. Zhou, H. Geng, Q. Chen, S. Kim, R. Zhang, C. Zhang, B. Chang, S. Li, H. Fu, L. Xue, H. Wang, W. Li, W. Chen, M. Gao, L. Ye, Y. Zhou, Y. Ouyang, C. Zhang, F. Gao, C. Yang, Y. Li, Z.-G. Zhang, *Nat. Commun.,* 2023, **14**, 2926.

[15] M. Lv, Q. Wang, J. Zhang, Y. Wang, Z. G. Zhang, T. Wang, H. Zhang, K. Lu, Z. Wei, D. Deng, *Adv. Mater.,* 2023, **36**, 2310046.

[16] C. Sun, J. W. Lee, Z. Tan, T. N. L. Phan, D. Han, H. G. Lee, S. Lee, S. K. Kwon, B. J. Kim, Y. H. Kim, *Adv. Energy Mater.,* 2023, **13**, 2301283.
